# Supplementary material for: Marine Biotechnology: Challenges and Development Market Trends for the Enhancement of Biotic Resources in Industrial Pharmaceutical and Food Applications. A Statistical Analysis of Scientific Literature and Business Models
Source: Mar Drugs. 2021 Jan 26;19(2):61. doi: 10.3390/md19020061 (PMC7912129; doi:10.3390/md19020061)
Supplement: Supplementary file 1 [file marinedrugs-19-00061-s001.pdf]

Challenge	Technology	Article title	Authors	Year
**Discovery**	**Biocatalysis and biosynthetis**	Biogenic synthesis and characterization of gold nanoparticles by a novel marine bacteria Marinobacter algicola: Progression from nanospheres to various geometrical shapes	Gupta R. & Padmanabhan P.	2018
Discovery	Biocatalysis and biosynthetis	Hydrosoluble Antioxidants by Enzymatic Glucosylation of a Vitamin E Derivative Using Marine α-d-Glucosidase from Aplysia fasciata	Tramice A., Andreotti G. et al.	2011
Discovery	Biocatalysis and biosynthetis	Purification and identification of novel angiotensin-I converting enzyme (ACE) inhibitory peptides from cultured marine microalgae (Nannochloropsis oculata) protein hydrolysate	Samarakoon K.W., O-Nam K. et al.	2013
Discovery	Biocatalysis and biosynthetis	Antioxidant capacity and prebiotic effects of Gracilaria neoagaro oligosaccharides prepared by agarase hydrolysis.	Zhang Y.H., Song X.N. et al.	2019
Discovery	Biocatalysis and biosynthetis	Biocatalytic resolution of glycidyl phenyl ether using a novel epoxide hydrolase from a marine bacterium, Rhodobacterales bacterium HTCC2654	Woo J.H., Kang J.H. et al.	2010
Discovery	Biocatalysis and biosynthetis	Exploration of the Glycosyltransferase BmmGT1 from a Marine-Derived bacillus strain as a potential enzyme tool for compound Glycol-Diversification	Liu Q., Ren P. et al.	2018
Discovery	Biocatalysis and biosynthetis	Biotransformation of bioactive (-)-mellein by a marine isolate of bacterium Stappia sp	Feng Z., Nenkep V. et al.	2010
Discovery	Biocatalysis and biosynthetis	Overexpression and characterization of lycopene cyclase (CrtY) from marine bacterium Paracoccus haeundaensis	Jeong, Hyug T. et al.	2013
Discovery	Biocatalysis and biosynthetis	Marine microorganisms as source of stereoselective esterases and ketoreductases: kinetic resolution of a prostaglandin intermediate.	De Vitis V., Guidi B. et al.	2015
Discovery	Biocatalysis and biosynthetis	α-Rhamnosidase activity in the marine isolate Novosphingobium sp. PP1Y and its use in the bioconversion of flavonoids	Izzo V., Tedesco P. et al.	2014
Discovery	**Biochemical and molecular characterization**	Marine L-Asparaginase: A novel microbial therapeutic approach for cancer	Venkata Siva Lakshmi T., Siva Mallika D. et al.	2015
Discovery	Biochemical and molecular characterization	Phenol, 2,4-bis(1,1-dimethylethyl) of marine bacterial origin inhibits quorum sensing mediated biofilm formation in the uropathogen Serratia marcescens	Padmavathi A.R., Abinaya B. et al.	2014
Discovery	Biochemical and molecular characterization	Evaluation and characterization of the plant growth promoting potentials of two heterocystous cyanobacteria for improving food grains growth	Suresh A., Soundararajan S. et al.	2019
Discovery	Biochemical and molecular characterization	Extraction, isolation, and characterization of alginate	Arunkumar K.	2017
Discovery	Biochemical and molecular characterization	Bioprospection of cellulolytic and lipolytic south atlantic deep-sea bacteria	Odisi E.J., Silvestrin M.B. et al.	2012
Discovery	Biochemical and molecular characterization	Biochemical characterization of a novel xylanase from Paenibacillus barengoltzii and its application in xylooligosaccharides production from corncobs	Liu X., Liu Y. et al.	2018
Discovery	Biochemical and molecular characterization	Aureolic acids from a marine-derived Streptomyces sp. WBF16	Lu J., Ma Y. et al.	2012
Discovery	Biochemical and molecular characterization	Diversity and chemical defense role of culturable non-actinobacterial bacteria isolated from the South China Sea gorgonians	Peng J., Zhang G. et al.	2013

Discovery	Biochemical and molecular characterization	Exopolysaccharides from extremophiles: From fundamentals to biotechnology	Nicolaus B., Kambourova M. et al.	2010
Discovery	Biochemical and molecular characterization	Marine biocatalysts: Enzymatic features and applications	Trincone A.	2011
Discovery	Biochemical and molecular characterization	Biotechnological potential of the seaweed Cladophora rupestris (Chlorophyta, Cladophorales) lipidic extract	Stabili M., Acquaviva M.V. et al.	2014
Discovery	Biochemical and molecular characterization	Acidophilic tannase from marine aspergillus awamori BTMFW032	Beena P.S., Soorej et al.	2010
Discovery	Biochemical and molecular characterization	Secondary metabolites of mangrove-associated strains of talaromyces	Nicoletti R., Salvatore M.M. et al.	2018
Discovery	Biochemical and molecular characterization	Natural products from polar organisms: Structural diversity, bioactivities and potential pharmaceutical applications	Tripathi V.C., Satish S. et al.	2018
Discovery	Biochemical and molecular characterization	Microbial exopolysaccharides	Mishra A. & Jha B.	2013
Discovery	Biochemical and molecular characterization	A review on chloro substituted marine natural product, chemical examination and biological activity	Kommu N., Rao Kundeti L.S. et al.	2019
Discovery	Biochemical and molecular characterization	Microalgae and cyanobacteria as enzyme biofactories	Brasil B.S.A.F, de Siqueira F.G. et al.	2017
Discovery	Biochemical and molecular characterization	Biodiscovery of new Australian thraustochytrids for production of biodiesel and long-chain omega-3 oils	Lee Chang K.Y., Dunstan G.A. et al.	2012
Discovery	Biochemical and molecular characterization	Molecular characterization and therapeutic potential of a marine bacterium Pseudoalteromonas sp. KMM 701 α-galactosidase	Balabanova L.A., Bakunina I.Y. et al.	2010
Discovery	Biochemical and molecular characterization	Production of cold-adapted enzymes by filamentous fungi from King George Island, Antarctica	Duarte A.W.F., Barato M.B. et al.	2018
Discovery	Biochemical and molecular characterization	Marine carbohydrate-based compounds with medicinal properties	Vasconcelos A.A. & Pomin V.H.	2018
Discovery	Biochemical and molecular characterization	Incorporation of Collagen from Marine Sponges (Spongin) into Hydroxyapatite Samples: Characterization and In Vitro Biological Evaluation	Parisi J.R., Fernandes K.R. et al.	2019
Discovery	Biochemical and molecular characterization	Bioactive compounds from marinemacroalgae and their hypoglycemic benefits	Zhao C., Yang C. et al.	2018
Discovery	Biochemical and molecular characterization	Molecular and biochemical characteristics of β-propeller phytase from marine Pseudomonas sp. BS10-3 and its potential application for animal feed additives	Nam S.J., Kim Y.O. et al.	2014
Discovery	Biochemical and molecular characterization	Halophilic and halotolerant actinomycetes from a marine saltern of Goa, India producing anti-bacterial metabolites	Ballav S., Kerkar S. et al.	2015
Discovery	Biochemical and molecular characterization	Biochemical characterization of an ulvan lyase from the marine flavobacterium Formosa agariphila KMM 3901T	Reisky L., Stanetty C. et al.	2018
Discovery	Biochemical and molecular characterization	Taxonomic and metabolite diversity of actinomycetes associated with three Australian ascidians	Buedenbender L., Carroll A.R. et al.	2017

Discovery	Biochemical and molecular characterization	Marine biosurfactants: biosynthesis, structural diversity and biotechnological applications	Kubicki S., Bollinger A. et al.	2019
Discovery	Biochemical and molecular characterization	Taxonomy, ecology and biotechnological applications of thraustochytrids: A review	Fossier Marchan L. , Lee Chang K. J. et al.	2018
Discovery	Biochemical and molecular characterization	Characterization of PL-7 Family Alginate Lyases From Marine Organisms and Their Applications	Inoue A.	2018
Discovery	Biochemical and molecular characterization	Gold from the sea: Marine compounds as inhibitors of the hallmarks of cancer	Schumacher M. Kelkel M. et al.	2011
Discovery	Biochemical and molecular characterization	A novel β-agarase with high pH stability from marine Agarivorans sp. LQ48	Long M., Yu Z. et al.	2010
Discovery	Biochemical and molecular characterization	Heparin-like entities from marine organisms	Colliec-Jouault S., Bavington C. et al.	2012
Discovery	Biochemical and molecular characterization	Marine-derived exopolysaccharides	Delbarre-Ladrat C., Boursicot V. et al.	2015
Discovery	Biochemical and molecular characterization	Sponge Chemical Diversity. From Biosynthetic Pathways to Ecological Roles	Genta-Jouve G. & Thomas O.P.	2012
Discovery	Biochemical and molecular characterization	Fatty acid profiling of new Irish microalgal isolates producing the high-value metabolites EPA and DHA	Archer L., Mc Gee D. et al.	2019
Discovery	Biochemical and molecular characterization	The large jellyfish Rhizostoma luteum as sustainable a resource for antioxidant properties, nutraceutical value and biomedical applications	Prieto L., Enrique-Navarro A. et al.	2018
Discovery	Biochemical and molecular characterization	American lobster Cathepsin D, an aspartic peptidase resistant to proteolysis and active in organic solvents, non-ionic detergents and salts	Rodriguez-Siordia I., Rojo-Arreola L. et al.	2018
Discovery	Biochemical and molecular characterization	Implications of extracellular polymeric substance matrices of microbial habitats associated with coastal aquaculture systems	Camacho-Chab J.C., Lango-Reynoso F. et al.	2016
Discovery	Biochemical and molecular characterization	The Marine Catenovulum agarivorans MNH15 and Dextranase: Removing dental plaque	Lai X., Liu X. et al.	2019
Discovery	Biochemical and molecular characterization	Epithermal neutron activation analysis in applied microbiology	Frontasyeva M. & Kirkesali E.	2012
Discovery	Biochemical and molecular characterization	New Source of 3D Chitin Scaffolds: The Red Sea Demosponge Pseudoceratina arabica (Pseudoceratinidae, Verongiida).	Shaala L.A., Asfour H.Z. et al.	2019
Discovery	Biochemical and molecular characterization	Cultivable bacterial community analysis of Saeu-Jeotgal, a Korean high-salt-fermented seafood, during ripening	Lee D.W., Jung G. et al.	2016
Discovery	Biochemical and molecular characterization	A novel glycosyl hydrolase family 16 β-agarase from the agar-utilizing marine bacterium gilvimarinus agarilyticus JEA5: The first molecular and biochemical characterization of agarase in genus gilvimarinus	Lee Y., Jo E. et al.	2018
Discovery	Biochemical and molecular characterization	Identification of a bioactive compound, violacein, from Microbulbifer sp. isolated from a marine sponge Hymeniacidon sinapium on the west coast of Korea	Won N.I., Lee G.E. et al.	2017
Discovery	Biochemical and molecular characterization	Discovery of a mcl-PHA with unexpected biotechnical properties: the marine environment of French Polynesia as a source for PHA-producing bacteria	Wecker P., Moppert X. et al.	2015

Discovery	Biochemical and molecular characterization	Fucoxanthine content in some black sea brown algae (Ochrophyta, phaeophyceae)	Tkachenko F.P. & Yakuba I.P.	2019
Discovery	Biochemical and molecular characterization	Porifera Lectins: Diversity, Physiological Roles and Biotechnological Potential.	Garderes J., Bourguet-Kondracki M.L. et al.	2015
Discovery	Biochemical and molecular characterization	Isolation and characterization of a novel agar-degrading marine bacterium, Gayadomonas joobiniege gen, nov, sp. nov., from the Southern Sea, Korea	Chi W.J., Park J.S. et al.	2013
Discovery	Biochemical and molecular characterization	Biochemical characterization of a novel cold-adapted GH39 β-agarase, AgaJ9, from an agar-degrading marine bacterium Gayadomonas joobiniege G7	Jung S., Lee C.R.et al.	2017
Discovery	Biochemical and molecular characterization	Biochemical characterization of a novel GH86 β-agarase producing neoagarohexaose from Gayadomonas joobiniege G7	Lee Y.R., Yung S. et al.	2018
Discovery	Biochemical and molecular characterization	Biochemical Characterization and Elucidation of Action Pattern of a Novel Polysaccharide Lyase 6 Family Alginate Lyase from Marine Bacterium Flammeovirga sp. NJ-04.	Li Q., Hu F. et al.	2019
Discovery	Biochemical and molecular characterization	Marine oligosaccharides originated from seaweeds: Source, preparation, structure, physiological activity and applications	Zhu B., Ni F. et al.	2020
Discovery	Biochemical and molecular characterization	Purification and characterization of a new κ-carrageenase from the marine bacterium vibrio sp. Nj-2	Zhu B. & Ning L.	2015
Discovery	Biochemical and molecular characterization	Characterization of an oleaginous unicellular green Microalga, Lobosphaera incisa (Reisigl, 1964) Strain K-1, isolated from a tidal flat in the Yellow Sea, Republic of Korea	Lee S., Lim S.R. et al.	2018
Discovery	Biochemical and molecular characterization	Disruption of N-acyl-homoserine lactone-specific signalling and virulence in clinical pathogens by marine sponge bacteria	Guetierrez-Barranquero J.A., Reen F.J. et al.	2019
Discovery	Biochemical and molecular characterization	Characterization and Applications of Marine Microbial Enzymes in Biotechnology and Probiotics for Animal Health	Nguyen T.H. & Nguyen V.D.	2017
Discovery	Biochemical and molecular characterization	Regulation of Extracellular Matrix Synthesis by Shell Extracts from the Marine Bivalve Pecten maximus in Human Articular Chondrocytes— Application for Cartilage Engineering	Bouyoucef M., Rakic R. et al.	2018
Discovery	Biochemical and molecular characterization	American Oyster, Crassostrea virginica, Expresses a Potent Antibacterial Histone H2B Protein	Seo J.K., Stephenson J. et al.	2010
Discovery	Biochemical and molecular characterization	β-1,3-glucanase inhibits activity of the killer toxin produced by the marine-derived yeast Williopsis saturnus WC91-2	Peng Y., Chi Z. et al.	2010
Discovery	Biochemical and molecular characterization	Purification, characterization and gene cloning of the killer toxin produced by the marine-derived yeast Williopsis saturnus WC91-2	Wang X.X., Chi Z. et al.	2012
Discovery	Biochemical and molecular characterization	Expression and characterization of a κ-carrageenase from marine bacterium Wenyingzhuangia aestuarii OF219: A biotechnological tool for the depolymerization of κ-carrageenan.	Shen J., Chang Y. et al.	2018
Discovery	Biochemical and molecular characterization	Expression and Characterization of a Novel β-Porphyranase from Marine Bacterium Wenyingzhuangia fucanilytica: A Biotechnological Tool for Degrading Porphyran.	Zhang Y., Chang Y. et al.	2019
Discovery	Biochemical and molecular characterization	Marine microorganisms as biocontrol agents against fungal phytopathogens and mycotoxins	Kong Q.	2018

Discovery	Biochemical and molecular characterization	A novel agaro-oligosaccharide-lytic β-galactosidase from Agarivorans gilvus WH0801.	Yang X., Liu Z. et al.	2018
Discovery	Biochemical and molecular characterization	Characterization of an α-agarase from Thalassomonas sp. LD5 and its hydrolysate	Zhang W., Xu J. et al.	2018
Discovery	Biochemical and molecular characterization	Screening of new cell cycle suppressive compounds from marine-derived microorganisms in Chinese hamster ovary cells	Kido M., Idogaki H. et al.	2020
Discovery	Biochemical and molecular characterization	Unusual glycosaminoglycans from a deep sea hydrothermal bacterium improve fibrillar collagen structuring and fibroblast activities in engineered connective tissues	Senni K., Gueniche F. et al.	2013
Discovery	Biochemical and molecular characterization	Culturable diversity and biochemical features of thraustochytrids from coastal waters of Southern China	Liu Y., Singh P. et al.	2014
Discovery	Biochemical and molecular characterization	First Report on Chitin in a Non-Verongiid Marine Demosponge: The Mycale euplectellioides Case.	Zoltowska-Aksamitowska S., Shaala L.A. et al.	2018
Discovery	Biochemical and molecular characterization	Isolation and molecular characterization of marine bacteria isolated from the beagle channel, Argentina	Cristobal H.A., Alvarenga A.E. et al.	2011
Discovery	Biochemical and molecular characterization	Natural flora and anticancer regime: Milestones and roadmap	Bhatnagar I., Thomas N.V. et al.	2013
Discovery	Biochemical and molecular characterization	Marine polysaccharide-based nanomaterials as a novel source of nanobiotechnological applications	Manivasagan P. & Oh J.	2016
Discovery	Biochemical and molecular characterization	Anti-HIV Activities of Marine Macroalgae	Vo T.S., Ngo D.H. et al.	2011
Discovery	Biochemical and molecular characterization	Marine collagen as a promising biomaterial for biomedical applications	Lim Y.S., Ok Y.J. et al.	2019
Discovery	Biochemical and molecular characterization	Blueprints for the Next Generation of Bioinspired and Biomimetic Mineralised Composites for Bone Regeneration.	Walsh P.J., Fee K. et al.	2018
Discovery	Biochemical and molecular characterization	Multi-scale thermal stability of a hard thermoplastic protein-based material	Latza V., Guerette P.A. et al.	2015
Discovery	Biochemical and molecular characterization	Marine polysaccharides for drug delivery in tissue engineering	Sarangi M.K., Rao M.E.B. et al.	2019
Discovery	Biochemical and molecular characterization	Phycobiliproteins: A novel green tool from marine origin blue-green algae and red algae	Chandra R., Parra R. et al.	2016
Discovery	Biochemical and molecular characterization	Biochemical characteristics and substrate degradation pattern of a novel Exo-Type β-Agarase from the Polysaccharide-Degrading Marine Bacterium Flammeovirga sp. Strain MY04	Han W., Chen Y. et al.	2016
Discovery	Biochemical and molecular characterization	Isolation and characterization of an eosinophilic GH 16 β-Agarase (AgaDL6) from an agar-degrading marine bacterium flammeovirga sp. HQM9	Liu Y., Tian X. et al.	2019
Discovery	Biochemical and molecular characterization	Biological active metabolite cyclo (l-Trp-l-Phe) produced by South China Sea sponge Holoxea sp. associated fungus Aspergillus versicolor strain TS08	Chu D., Peng C. et al.	2011
Discovery	Biochemical and molecular characterization	Substrate selection of adenylation domains for nonribosomal peptide synthetase (NRPS) in bacillamide C biosynthesis by marine Bacillus atrophaeus C89	Zhang F., Wang Y. et al.	2018

Discovery	Biochemical and molecular characterization	Microbial diversity in freshwater ecosystems and its industrial potential	Sharma P., Slathia P.S. et al.	2019
Discovery	Biochemical and molecular characterization	Biotechnological applications of scyphomedusae	Merquiol L., Romano G. et al.	2019
Discovery	Biochemical and molecular characterization	Marine microalgae with anti-cancer properties	Martinez Andradez K.A., Lauritano C. et al.	2018
Discovery	Biochemical and molecular characterization	Potent inhibition of monoamine oxidase b by a piloquinone from marine-derived streptomyces sp. CNQ-027	Lee H.W., Choi H. et al.	2017
Discovery	Biochemical and molecular characterization	Freeze-dried microalgae of Nannochloropsis oculata improve soybean oil's oxidative stability	Lee Y.L., Chuang Y.C. et al.	2013
Discovery	Biochemical and molecular characterization	Occurrence and biosynthesis of carotenoids in phytoplankton	Huang J.J. Lin S. et al.	2017
Discovery	Biochemical and molecular characterization	Solution structure of polytheonamide B, a highly cytotoxic nonribosomal polypeptide from marine sponge.	Hamada T., Matsunaga S. et al.	2010
Discovery	Biochemical and molecular characterization	Industrial applications of marine carbohydrates	Sudha P.N., Aisverya S. et al.	2014
Discovery	Biochemical and molecular characterization	Properties and biotechnological applications of natural and engineered haloalkane dehalogenases	Nagata Y., Ohtsubo Y. et al.	2015
Discovery	Biochemical and molecular characterization	Characterization of a novel marine unicellular alga, Pseudoneochloris sp. strain NKY372003 as a high carbohydrate producer	Aketo T., Hashizume R. et al.	2020
Discovery	Biochemical and molecular characterization	Algal polysaccharides and health	Misurcova L., Orsavová J. et al.	2015
Discovery	Biochemical and molecular characterization	Characterization of amylase produced by cold-adapted bacteria from Antarctic samples	Ottoni J.R., Rodrigues Silva T. et al.	2020
Discovery	Biochemical and molecular characterization	The hydrocarbon-degrading marine bacterium Cobetia sp. strain MM1IDA2H-1 produces a biosurfactant that interferes with quorum sensing of fish pathogens by signal hijacking	Ibacache-Quiroga C., Ojeda J. et al.	2013
Discovery	Biochemical and molecular characterization	Polar lipid profile of Saccharina latissima, a functional food from the sea	Rey F., Lopes D. et al.	2019
Discovery	Biochemical and molecular characterization	Biotechnological potential of benthic marine algae collected along the Brazilian coast	Martins A.P., Zambotti-Villela L. et al.	2018
Discovery	Biochemical and molecular characterization	Cyanoflan: A cyanobacterial sulfated carbohydrate polymer with emulsifying properties	Mota R., Vidal R. et al.	2020
Discovery	Biochemical and molecular characterization	Nutritional value of selected macroalgae	Patarra R.F., Paiva L. et al.	2011
Discovery	Biochemical and molecular characterization	Marine-derived fungi: Diversity of enzymes and biotechnological applications	Bonugli-Santos R.C., Vasconcelos M.R.D.S. et al.	2015
Discovery	Biochemical and molecular characterization	Nannochloropsis sp. and Spirulina sp. as a Source of Antifungal Compounds to Mitigate Contamination by Fusarium graminearum Species Complex	Scaglioni P.T., Pagnussatt F.A et al.	2019

Discovery	Biochemical and molecular characterization	Glycolipids from seaweeds and their potential biotechnological applications.	Plouguerné E., da Gama B.A.P. et al.	2014
Discovery	Biochemical and molecular characterization	The mechanically adaptive connective tissue of echinoderms: Its potential for bio-innovation in applied technology and ecology	Barbaglio A., Tricarico S. et al.	2012
Discovery	Biochemical and molecular characterization	Marine-derived collagen biomaterials from echinoderm connective tissues	Ferrario C., Leggio L. et al.	2017
Discovery	Biochemical and molecular characterization	Sterol and steroid catabolites from cholesterol produced by the psychrophile Pseudoalteromonas haloplanktis	Gelzo M., Lamberti A. et al.	2014
Discovery	Biochemical and molecular characterization	Anti-biofilm activity of the Antarctic marine bacterium Pseudoalteromonas haloplanktis TAC125	Papa R., Parrilli E. et al.	2013
Discovery	Biochemical and molecular characterization	Thermal properties of an exopolysaccharide produced by a marine thermotolerant Bacillus licheniformis by ATR-FTIR spectroscopy	Caccamo M.T., Gugliandolo C. et al.	2020
Discovery	Biochemical and molecular characterization	Production and Biotechnological Potential of Extracellular Polymeric Substances from Sponge-Associated Antarctic Bacteria.	Caruso C., Rizzo C. et al.	2018
Discovery	Biochemical and molecular characterization	Marine Fungi from the Sponge Grantia compressa: Biodiversity, Chemodiversity, and Biotechnological Potential.	Bovio E., Garzoli L. et al.	2019
Discovery	Biochemical and molecular characterization	Marine polysaccharides in microencapsulation and application to aquaculture: "From sea to sea"	Borgogna M., Bellich B. et al.	2011
Discovery	Biochemical and molecular characterization	New emulsifying and cryoprotective exopolysaccharide from Antarctic Pseudomonas sp. ID1	Carrion O., Delgado L. et al.	2015
Discovery	Biochemical and molecular characterization	Antibacterial activity of a sulfated galactan extracted from the marine alga Chaetomorpha aerea against Staphylococcus aureus	Pierre G., Sopena V. et al.	2011
Discovery	Biochemical and molecular characterization	Diversity and bioactive potential of endospore-forming bacteria cultured from the marine sponge Haliclona simulans	Phelan R.W., O'Halloran J.A. et al.	2012
Discovery	Biochemical and molecular characterization	Isolation, Characterization and Biotechnological Potentials of Thraustochytrids from Icelandic Waters.	Stefansson M.O., Baldursson S. et al.	2019
Discovery	Biochemical and molecular characterization	Bursatella leachii from Mar Menor as a Source of Bioactive Molecules: Preliminary Evaluation of the Nutritional Profile, In Vitro Biological Activities, and Fatty Acids Contents	Braga T., Rodrigues M.J. et al.	2017
Discovery	Biochemical and molecular characterization	Haloarchaeal carotenoids: Healthy novel compounds from extreme environments	Giani M., Garbayo I. et al.	2019
Discovery	Biochemical and molecular characterization	The unique mechanistic transformations involved in the biosynthesis of modular natural products from marine cyanobacteria	Jones A.C., Monroe E.A. et al.	2010
Discovery	Biochemical and molecular characterization	Diversity, biological roles and biosynthetic pathways for sugar-glycerate containing compatible solutes in bacteria and archaea	Empadinhas N. & Costa M.S.	2011
Discovery	Biochemical and molecular characterization	A revision on the red alga Dilsea Carnosa	Marques J.F. & Pereira L.	2016
Discovery	Biochemical and molecular characterization	Thermostable and Alkaline Cellulases from Marine Sources	Annamalai N., Rajeswari M.V. et al.	2016

Discovery	Biochemical and molecular characterization	Marine microbes as a valuable resource for brand new industrial biocatalysts	Beygmoradi A. & Homaei A.	2017
Discovery	Biochemical and molecular characterization	Extraction and purification of a highly thermostable alkaline caseinolytic protease from wastes Penaeus vannamei suitable for food and detergent industries	Dadshahi Z., Homaei A. et al.	2016
Discovery	Biochemical and molecular characterization	Marine microbial L-asparaginase: Biochemistry, molecular approaches and applications in tumor therapy and in food industry	Qeshmi F.I., Homaei A. et al.	2018
Discovery	Biochemical and molecular characterization	Sources of marine superoxide dismutases: Characteristics and applications	Zeinali F., Homaei A. et al.	2015
Discovery	Biochemical and molecular characterization	Diversity and industrial potential of hydrolaseproducing halophilic/halotolerant eubacteria	Setati M.E.	2010
Discovery	Biochemical and molecular characterization	Isolation and characterization of macromolecular protein R-Phycoerythrin from Portieria hornemannii	Senthilkumar N., Suresh V. et al.	2013
Discovery	Biochemical and molecular characterization	Drug delivery systems and cartilage tissue engineering scaffolding using marine-derived products	Silva T.H. & Reis R.L.	2015
Discovery	Biochemical and molecular characterization	Finding new enzymes from bacterial physiology: A successful approach illustrated by the detection of novel oxidases in Marinomonas mediterranea	Sanchez-Amat A., Solano F. et al.	2010
Discovery	Biochemical and molecular characterization	An evaluation of the genus Amphidinium (Dinophyceae) combining evidence from morphology, phylogenetics, and toxin production, with the introduction of six novel species	Karafs S., Teng S.T. et al.	2017
Discovery	Biochemical and molecular characterization	Review: Bioactive marine drugs and marine biomaterials for brain diseases	Grosso C., Valentao P. et al.	2014
Discovery	Biochemical and molecular characterization	Cyanobactins from cyanobacteria: Current genetic and chemical state of knowledge	Martins J. & Vasconcelos V.	2015
Discovery	Biochemical and molecular characterization	Marine extremophiles a source of hydrolases for biotechnological applications	Dalmaso G.Z.L., Ferreira D. et al.	2015
Discovery	Biochemical and molecular characterization	Bioactive composite films with chitosan and carotenoproteins extract from blue crab shells: Biological potential and structural, thermal, and mechanical characterization	Hamdi M., Nasri R. et al.	2019
Discovery	Biochemical and molecular characterization	Chapter 18: Marine Metabolites: Oceans of Opportunity	Gallimore W.	2017
Discovery	Biochemical and molecular characterization	Antimicrobial peptides from marine invertebrates: Challenges and perspectives in marine antimicrobial peptide discovery	Sperstad S.V., Haug T. et al.	2011
Discovery	Biochemical and molecular characterization	Marine biotechnology: Focus on anticancer drugs	Rastogi A., Ruqiua S. et al.	2016
Discovery	Biochemical and molecular characterization	A review on trend of marine sources for the development of functional foods	Bajpai V.K.	2017
Discovery	Biochemical and molecular characterization	A Review of Pigments Derived from Marine Natural Products	Ye K.X., Fan T.T. et al.	2019
Discovery	Biochemical and molecular characterization	Wewakamide a and guineamide g, cyclic depsipeptides from the marine cyanobacteria lyngbya semiplena and lyngbya majuscula	Han B., Gross H. et al.	2011

Discovery	**Bioinformatics**	Marine Bacterial Compounds Evaluated by In Silico Studies as Antipsychotic Drugs Against Schizophrenia	Thiyagarajamoorthy D.K., Arulanandam C.D. et al.	2018
Discovery	Bioinformatics	In silico biological activity of steroids from the marine gastropods Telescopium telescopium collected from south west Coast of India	Ragi A.S., Leena P.P. et al.	2018
Discovery	Bioinformatics	Prospecting biotechnologically-relevant monooxygenases from cold sediment metagenomes: An in silico approach	Masumeci M.A., Lozada M. et al.	2017
Discovery	Bioinformatics	Emerging concepts promising new horizons for marine biodiscovery and synthetic biology	Reen F.J., Gutiérrez-Barranquero J.A. et al.	2015
Discovery	Bioinformatics	An in silico based comparison of drug interactions in wild and mutant human β-tubulin through docking studies	Chellasamy S. & Mohammed S.M.M.	2014
Discovery	Bioinformatics	Biochemical characterization of a family 15 carbohydrate esterase from a bacterial marine Arctic metagenome	De Santi C., Willassen N.P. et al.	2016
Discovery	Bioinformatics	AhlX, an N-acylhomoserine Lactonase with Unique Properties	Liu P., Chen Y. et al.	2019
Discovery	**Chemical synthesis**	Preparation of well-dispersed chitosan/alginate hollow multilayered microcapsules for enhanced cellular internalization	Ribeiro C., Borges J. et al.	2018
Discovery	Chemical synthesis	Marine polysaccharides in pharmaceutical applications: An overview	Laurienzo P.	2010
Discovery	Chemical synthesis	Melanin and melanin-related polymers as materials with biomedical and biotechnological applications— Cuttlefish ink and mussel foot proteins as inspired biomolecules	Solano F.	2017
Discovery	**Drug discovery**	Blue bugs for red biotechnology	Jagadeesan Y. & Balaiah A.	2015
Discovery	Drug discovery	Algal biotechnology: An update from industrial and medical point of view	Shanmugam H., Sathasivam R. et al.	2018
Discovery	Drug discovery	Chapter 18: Biotechnological aspects of mangrove microorganisms	Singh Y.D., Singh M.C. et al.	2020
Discovery	Drug discovery	Marine Viruses: the Beneficial Side of a Threat	Sanchez-Paz A., Muhlia-Almazan A. et al.	2014
Discovery	Drug discovery	Marine Microbial Secondary Metabolites: Pathways, Evolution and Physiological Roles	Giordano D., Coppola D. et al.	2015
Discovery	Drug discovery	Enzymatic processes in marine biotechnology	Trincone A.	2017
Discovery	Drug discovery	Cnidarian Interaction with Microbial Communities: From Aid to Animal's Health to Rejection Responses.	Stabili L., Parisi M.G. et al.	2018
Discovery	Drug discovery	Marine algicolous endophytic fungi-a promising drug resource of the era	Sarasan M., Puthumana J. et al.	2017
Discovery	Drug discovery	Biotechnology of marine fungi	Damare S., Singh P. et al.	2011
Discovery	Drug discovery	Developments of cyanobacteria for nano-marine drugs: Relevance of nanoformulations in cancer therapies	Bajpai V.K., Shukla S. et al.	2018
Discovery	Drug discovery	Industrial applications of marine polysaccharides	Joshi S.N., Bedekar A.N. et al.	2013
Discovery	Drug discovery	The place for enzymes and biologically active peptides from marine organisms for application in industrial and pharmaceutical biotechnology	Morlighem J.E.R.L. & Radis-Baptista G.	2019

Discovery	Drug discovery	Natural marine sponges for bone tissue engineering: The state of art and future perspectives	Granito R.N., Custódio M.R. et al.	2017
Discovery	Drug discovery	New marine natural products from sponges (Porifera) of the order Dictyoceratida (2001 to 2012); a promising source for drug discovery, exploration and future prospects	Mehbub M.F., Perkins M.V.et al.	2016
Discovery	Drug discovery	Hypotensive, hypoglycaemic and hypolipidaemic effects of bioactive compounds from microalgae and marine micro-organisms	Zhao C., Wu Y. et al.	2015
Discovery	Drug discovery	From discovery to production: Biotechnology of marine fungi for the production of new antibiotics	Silber J., Kramer A. et al.	2016
Discovery	Drug discovery	Biotechnological implications of hydrolytic enzymes from marine microbes	Vashist P., Kanchana R. et al.	2019
Discovery	Drug discovery	Marine natural products: A lead for anti-cancer	Sarfaraj H.M., Sheeba F. et al.	2012
Discovery	Drug discovery	Marine Natural Products from New Caledonia-A Review	Motuhi S.E., Mehiri M. et al.	2016
Discovery	Drug discovery	Future prospects and health benefits of functional ingredients from marine bio-resources: A review	Samarakoon K.W., Elvitigala D.A.S. et al.	2014
Discovery	Drug discovery	Bioactive compounds in seaweeds: An overview of their biological properties and safety	Rengasamy K.R.R., Mahomoodaly M.F. et al.	2020
Discovery	Drug discovery	Marine sponge-associated bacteria as a potential source for polyhydroxyalkanoates.	Sathiyanarayanan G., Saibaba G. et al.	2017
Discovery	Drug discovery	Novel insights on the symbiotic interactions of marine sponge-associated microorganisms: Marine microbial biotechnology perspective	Bramhachari P.V., Mutyala S. et al.	2016
Discovery	Drug discovery	Aquaculture: An overview of chemical ecology of seaweeds (food species) in natural products	Gnanavel V., Roopan S.M. et al.	2019
Discovery	Drug discovery	Versatile biological macromolecules: Chitin and chitosan	Garg N. & Kashyap S.R.	2014
Discovery	Drug discovery	Tiny microbes with a big impact: The role of cyanobacteria and their metabolites in shaping our future	Mazard S., Panesyan A. et al.	2016
Discovery	Drug discovery	Application of molecular genetic and microbiological techniques in ecology and biotechnology of cyanobacteria	Koksharova O.A.	2010
Discovery	Drug discovery	Jellyfish-Associated Microbiome in the Marine Environment: Exploring Its Biotechnological Potential.	Tinta T., Kogovsek T. et al.	2019
Discovery	Drug discovery	Biotechnological potential of Korean marine microalgal strains and its future prospectives	Hong J.W., Kang N.S. et al.	2019
Discovery	Drug discovery	Marine algae: A source of biomass for biotechnological applications	Stengel D.B. & Connan S.	2015
Discovery	Drug discovery	Algal chemodiversity and bioactivity: sources of natural variability and implications for commercial application.	Stengel D.B., Connan S. et al.	2011
Discovery	Drug discovery	Bio-products produced by marine yeasts and their potential applications	Chi Z., Liu G.L. et al.	2016
Discovery	Drug discovery	Biology and Industrial Applications of Chlorella: Advances and Prospects.	Liu J. & Chen F.	2016
Discovery	Drug discovery	Rhamnolipid biosurfactants: Evolutionary implications, applications and future prospects from untapped marine resource	Kiran G.S., Ninawe A.S. et al.	2016

Discovery	Drug discovery	Marine Spongin: Naturally Prefabricated 3D Scaffold-Based Biomaterial.	Jesionowski T., Norman M. et al.	2018
Discovery	Drug discovery	Industry perspectives of marine-derived proteins as biomaterials	Kim S.K., Ngo D.H. et al.	2013
Discovery	Drug discovery	Marine Microalgae Biotechnology: Present Trends and Future Advances. Present Trends and Future Advances	Venkatesan J., Manivasagan P. et al.	2015
Discovery	Drug discovery	Research and application of marine microbial enzymes: Status and prospects	Zhang C. & Kim S.K.	2010
Discovery	Drug discovery	Application of marine biomaterials for nutraceuticals and functional foods	Zhang C., Li X. et al.	2012
Discovery	Drug discovery	Marine derived pharmaceuticals- development of natural health products from marine biodiversity	Murti Y. & Agrawal T.	2010
Discovery	Drug discovery	Marine organisms with anti-diabetes properties	Lauritano C. & Ianora A.	2016
Discovery	Drug discovery	Microalgal enzymes with biotechnological applications	Vingiani G.M., De Luca P. et al.	2019
Discovery	Drug discovery	Manzamines: A potential for novel cures	Radwan M., Hanora A. et al.	2012
Discovery	Drug discovery	Marine polysaccharides: A source of bioactive molecules for cell therapy and tissue engineering	Senni K., Pereira J. et al.	2011
Discovery	Drug discovery	Evolving marine biomimetics for regenerative dentistry.	Green D.W., Lai W.F. et al.	2014
Discovery	Drug discovery	Biotechnological Applications of Marine Enzymes From Algae, Bacteria, Fungi, and Sponges.	Parte S., Sirisha V.L. et al.	2017
Discovery	Drug discovery	Marine enzymes and their industrial and biotechnological applications	Dumorné K. & Severe R.	2018
Discovery	Drug discovery	Advances in antarctic research for antimicrobial discovery: A comprehensive narrative review of bacteria from antarctic environments as potential sources of novel antibiotic compounds against human pathogens and microorganisms of industrial importance	Nunez-Montero K., Barrientos L. et al.	2018
Discovery	Drug discovery	Recent Advances in Marine Enzymes for Biotechnological Processes	Lima R.N. & Porto A.L.M.	2016
Discovery	Drug discovery	Marine drugs for cancer: Surfacing biotechnological innovations from the oceans	Jimenez P.C., Wilke D.V. et al.	2018
Discovery	Drug discovery	Biotechnological potential of sponge-associated bacteria	Santos-Gandelman J.F., Giambiagi-deMarval M. et al.	2014
Discovery	Drug discovery	Application of marine polysaccharides in nanotechnology	Melo-Silveira R.F., Almeida-Lima J. et al.	2013
Discovery	Drug discovery	The hidden biotechnological potential of marine invertebrates: The Polychaeta case study	Rodrigo A.P. & Costa P.M.	2019
Discovery	Drug discovery	Microbial diseases of bivalve mollusks: Infections, immunology and antimicrobial defense	Zannella C., Mosca F. et al.	2017
Discovery	Drug discovery	Marine sponges - molecular biology and biotechnology	Jackson S.A., Kennedy J. et al.	2015
Discovery	Drug discovery	Review of marine algae as source of bioactive metabolites: A marine biotechnology approach	Carvalho L.G. & Pereira L.	2014
Discovery	Drug discovery	Bacterial exopolysaccharides: Functionality and prospects	Nwodo U.U., Green E. et al.	2012

Discovery	Drug discovery	New insights on the marine cytochrome P450 enzymes and their biotechnological importance	Sharifian S., Homaei A. et al.	2020
Discovery	Drug discovery	Microorganisms living on macroalgae: Diversity, interactions, and biotechnological applications	Martin M., Portetelle D. et al.	2014
Discovery	Drug discovery	Marine-derived Bioactive Peptides: Their Cardioprotective Activities and Potential Applications	Vijayakumar M., Noorlidah A. et al.	2013
Discovery	Drug discovery	Marine yeast isolation and industrial application.	Zaky A.S., Tucker G.A. et al.	2014
Discovery	Drug discovery	Bioactive compounds isolated from neglected predatory marine gastropods	Turner A.K., Craik D.J. et al.	2018
Discovery	Drug discovery	Food ingredients from the marine environment. Marine biotechnology meets food science and technology	Boziaris I.S.	2014
Discovery	Drug discovery	Health benefits of marine foods and ingredients	Larsen R., Eilertsen K.E. et al.	2011
Discovery	Drug discovery	Importance of marine thermophiles in biotechnological applications	Mathai S., Roy K.R. et al.	2014
Discovery	Drug discovery	Marine natural flora: A potent source of anticancer metabolites	Shukla S. & Kim M.	2016
Discovery	**Omic technologies**	A novel esterase from a marine metagenomic library exhibiting salt tolerance ability	Fang Z., Li J. et al.	2014
Discovery	Omic technologies	Functional marine metagenomic screening for anti-quorum sensing and anti-biofilm activity	Yaniv K., Golberg K. et al.	2017
Discovery	Omic technologies	Photoprotective bioactivity present in a unique marine bacteria collection from Portuguese deep sea hydrothermal vents	Martins A., Tenreiro T. et al.	2013
Discovery	Omic technologies	Oxygenated elansolid-type of polyketide spanned macrolides from a marine heterotrophic Bacillus as prospective antimicrobial agents against multidrug-resistant pathogens	Kizhakkekalam V.K., Chakraborty K. et al.	2020
Discovery	Omic technologies	Marine Enzymes: Production and Applications for Human Health.	Rao T.E., Imchen M. et al.	2017
Discovery	Omic technologies	Drugs and leads from the ocean through biotechnology	Paniagua-Michel J.D.J., Olmos-Soto J. et al.	2015
Discovery	Omic technologies	Structural and functional analysis of a low-temperature-active alkaline esterase from South China Sea marine sediment microbial metagenomic library	Hu Y., Liu Y. et al.	2015
Discovery	Omic technologies	Genome sequencing and analyses of two marine fungi from the North Sea unraveled a plethora of novel biosynthetic gene clusters	Kumar A., Sorensen J.L. et al.	2018
Discovery	Omic technologies	The anemonia viridis venom: Coupling biochemical purification and rna-seq for translational research	Nicosia A., Vikov A. et al.	2018
Discovery	Omic technologies	Biotechnological Applications of Bioactive Peptides From Marine Sources	Giordano D., Costantini M. et al.	2018
Discovery	Omic technologies	Antimicrobial compounds from seaweeds-associated bacteria and fungi	Singh R.P., Kumari P. et al.	2015
Discovery	Omic technologies	A cold-adapted carbohydrate esterase from the oil-degrading marine Bacterium Microbulbifer thermotolerans DAU221: Gene cloning, purification, and characterization	Lee Y.S., Heo J.B. et al.	2014
Discovery	Omic technologies	Entotheonella Bacteria as Source of Sponge-Derived Natural Products: Opportunities for Biotechnological Production	Bhushan A., Peters E.E. et al.	2017

Discovery	Omic technologies	An environmental bacterial taxon with a large and distinct metabolic repertoire	Wilson M.C., Mori T. et al.	2014
Discovery	Omic technologies	The holo-transcriptome of the zoantharian protopalythoa variabilis (cnidaria: Anthozoa): A plentiful source of enzymes for potential application in green chemistry, industrial and pharmaceutical biotechnology	Morlighem J.E.R.L., Huang C. et al.	2018
Discovery	Omic technologies	The biotechnological potential of marine bacteria in the novel lineage of Pseudomonas pertucinogena	Bollinger A., Thies S. et al.	2020
Discovery	Omic technologies	Bio-mining the microbial treasures of the ocean: New natural products	Imhoff J.F., Labes A. et al.	2011
Discovery	Omic technologies	Characterization of a Non-fibrillar-Related Collagen in the Mollusc Haliotis tuberculata and its Biological Activity on Human Dermal Fibroblasts	Fleury C., Serpentini A. et al.	2011
Discovery	Omic technologies	Transcriptomic analysis reveals the wound healing activity of Mussel Myticin C	Rey-Campos M., Moreira R. et al.	2020
Discovery	Omic technologies	Metagenomic studies of the Red Sea	Behzad H., Ibarra M.A. et al.	2016
Discovery	Omic technologies	Proteomics meets blue biotechnology: A wealth of novelties and opportunities	Hartmann E.M., Durighello E. et al.	2014
Discovery	Omic technologies	Chondroitin Lyase from a Marine Arthrobacter sp. MAT3885 for the Production of Chondroitin Sulfate Disaccharides	Kale V., Friðjónsson O. et al.	2015
Discovery	Omic technologies	Biotechnological Exploitation of Marine Animals	Das S.	2013
Discovery	Omic technologies	Emerging strategies and integrated systems microbiology technologies for biodiscovery of marine bioactive compounds	Rocha-Martin J., Harrington C. et al.	2014
Discovery	Omic technologies	Isolation and characterization of the gene cluster for biosynthesis of the thiopeptide antibiotic TP-1161	Engelhardt K., Degnes K.F. et al.	2010
Discovery	Omic technologies	Comparative genomics and CAZyme genome repertoires of marine Zobellia amurskyensis KMM	Chernysheva N., Bystritskaya E. et al.	2019
Discovery	Omic technologies	Functional gene-based discovery of phenazines from the actinobacteria associated with marine sponges in the South China Sea	Karuppiah V., Li Y. et al.	2015
Discovery	Omic technologies	De novo transcriptome of the cosmopolitan dinoflagellate Amphidinium carterae to identify enzymes with biotechnological potential	Lauritano C., De Luca D. et al.	2017
Discovery	Omic technologies	Marine fungi: Biotechnological perspectives from deep-hypersaline anoxic basins	Barone G., Varrella S. et al.	2019
Discovery	Omic technologies	Bioinformatics for Marine Products: An Overview of Resources, Bottlenecks, and Perspectives.	Ambrosino L., Tangherlini M. et al.	2019
Discovery	Omic technologies	Marine natural products from microalgae: An -omics overview	Lauritano C., Ferrante M.I. et al.	2019
Discovery	Omic technologies	Marine microorganisms as a promising and sustainable source of bioactive molecules	Romano G., Costantini M. et al.	2017
Discovery	Omic technologies	Omics approaches in marine biotechnology: The treasure of ocean for human betterments	Abid F., Zahid M.A. et al.	2018
Discovery	Omic technologies	Marine actinomycetes: An ongoing source of novel bioactive metabolites	Subramani R. & Aalbersberg W.	2012
Discovery	Omic technologies	Properties and Applications of Extremozymes from Deep-Sea Extremophilic Microorganisms: A Mini Review.	Jin M., Gai Y. et al.	2019
Discovery	Omic technologies	Genomic approaches in marine biodiversity and aquaculture	Huete-Perez J.A. & Quezada F.	2013

Discovery	Omic technologies	Bacterial diversity studied by next-generation sequencing in a mature phototrophic Navicula sp-based biofilm promoted into a shrimp culture system	Martinez-Cordova L.R., Martínez-Porchas M. et al.	2017
Discovery	Omic technologies	Quantification of the Genetic Expression of bgl-A, bgl, and CspA and Enzymatic Characterization of β-Glucosidases from Shewanella sp. G5	Cristobal H.A., Poma H.R. et al.	2016
Discovery	Omic technologies	Enzymes from Marine Polar Regions and Their Biotechnological Applications.	Bruno S., Coppola D. et al.	2019
Discovery	Omic technologies	Biotechnologies from marine bivalves	Venier P., Gerdol M. et al.	2018
Discovery	Omic technologies	Marine microorganisms: Potential application and challenges	Baharum S.N., Beng E.K. et al.	2010
Discovery	Omic technologies	Integrated (Meta) genomic and synthetic biology approaches to develop new biocatalysts	Parages M.L., Gutiérrez-Barranquero J.A. et al.	2016
Discovery	Omic technologies	Biotechnological Potential of Cold Adapted Pseudoalteromonas spp. Isolated from 'Deep Sea' Sponges.	Borchert E., Knobloch S. et al.	2017
Discovery	Omic technologies	Valorisation of the microalgae Nannochloropsis gaditana biomass by proteomic approach in the context of circular economy	Fernandez-Acero F.J., Amil-Ruiz F. et al.	2019
Discovery	Omic technologies	Integrating mass spectrometry and genomics for cyanobacterial metabolite discovery	Moss N.A., Bertin M.J. et al.	2016
Discovery	Omic technologies	Use of genome-scale models to get new insights into the marine actinomycete genus Salinispora	Contador C.A., Rodriguez V. et al.	2019
Discovery	Omic technologies	Marine natural products: A new wave of drugs?	Montaser R. & Luesch H.	2011
Discovery	Omic technologies	Cyanobacterial genomics for ecology and biotechnology	Hess W.R.	2011
Discovery	Omic technologies	UPLC-MSE profiling of Phytoplankton metabolites: application to the identification of pigments and structural analysis of metabolites in Porphyridium purpureum.	Juin C., Bonnet A. et al.	2015
Discovery	Omic technologies	Exploring the diversity and metabolic potential of actinomycetes from temperate marine sediments from Newfoundland, Canada	Duncan K.R., Haltli B. et al.	2015
Discovery	Omic technologies	Cloning and characterization of a novel cold-active glycoside hydrolase family 1 enzyme with β-glucosidase, β-fucosidase and β-galactosidase activities	Wierzbicka-Wos A., Bartasun P. et al.	2013
Discovery	Omic technologies	Isolation and characterization of a GDSL esterase from the metagenome of a marine sponge-associated bacteria	Okamura Y., Kimura T. et al.	2010
Discovery	Omic technologies	Mycoepoxydiene suppresses HeLa cell growth by inhibiting glycolysis and the pentose phosphate pathway	Jin K., Li L. et al.	2017
Discovery	Omic technologies	Bioinformatic techniques on marine genomics	Bilal A.M., Sijjad H.M. et al.	2015
Discovery	Omic technologies	Stress-driven discovery of a cryptic antibiotic produced by Streptomyces sp. WU20 from Kueishantao hydrothermal vent with an integrated metabolomics strategy	Shi Y., Pan C. et al.	2017
Discovery	**Optimization of culture conditions**	Production of Bacillus subtilis-fermented red alga Porphyra dentata suspension with fibrinolytic and immune-enhancing activities	Lin H.T.V., Hwang P.A. et al.	2014
Discovery	Optimization of culture conditions	Marine enzymes production tools to the pharmaceutical industry	Abdul B.A.A., Alijani S. et al.	2019
Discovery	Optimization of culture conditions	Pseudoalteromonas haloplanktis produces methylamine, a volatile compound active against Burkholderia cepacia complex strains	Sannino F., Parrilli E. et al.	2017

Discovery	**Optimization of harvesting and extraction methods**	Protective role of antioxidants capacity of Hyrtios aff. Erectus sponge extract against mixture of persistent organic pollutants (POPs)-induced hepatic toxicity in mice liver: biomarkers and ultrastructural study	Adb El-Moneam N.M., Shreadah M.A. et al.	2017
Discovery	Optimization of harvesting and extraction methods	Characterization of two anti-fungal lipopeptides produced by Bacillus amyloliquefaciens SH-B10	Chen L., Wang N. et al.	2010
Discovery	Optimization of harvesting and extraction methods	A novel red pigment from marine Arthrobacter sp. G20 with specific anticancer activity	Afra S., Makhdoumi A. et al.	2017
Discovery	Optimization of harvesting and extraction methods	Antioxidant Peptide Purified from Enzymatic Hydrolysates of Isochrysis Zhanjiangensis and Its Protective Effect against Ethanol Induced Oxidative Stress of HepG2 Cells	Chen M.F., Zhang Y.Y. et al.	2019
Discovery	Optimization of harvesting and extraction methods	Photoprotective substances derived from marine algae	Pangestuti R., Siahaan E.A. et al.	2018
Discovery	Optimization of harvesting and extraction methods	Mode of action of diterpene and characterization of related metabolites from the soft coral, Xeniaelongata	Adrianasolo E.H., Haramaty L. et al.	2014
Discovery	Optimization of harvesting and extraction methods	Preliminary characterization, antioxidant properties and production of chrysolaminarin from marine diatom Odontella aurita.	Xia S., Gao B. et al.	2014
Discovery	Optimization of harvesting and extraction methods	HPLC method for microanalysis and pharmacokinetics of marine sulfated polysaccharides, propylene glycol alginate sodium sulfate	Lee Y.T., Li C.X. et al.	2015
Discovery	Optimization of harvesting and extraction methods	Inhibitory effects of Streptomyces sp. MBTH32 Metabolites on Sortase A and Sortase A-Mediated Cell Clumping of Staphylococcus aureus to Fibrinogen	Chung B., Kwon O.S. et al.	2019
Discovery	Optimization of harvesting and extraction methods	First identification of marine diatoms with anti-tuberculosis activity	Lauritano C., Martin J. et al.	2018
Discovery	Optimization of harvesting and extraction methods	Decoding bioactive polar lipid profile of the macroalgae Codium tomentosum from a sustainable IMTA system using a lipidomic approach	Da Costa E., Melo T. et al.	2015
Discovery	Optimization of harvesting and extraction methods	Investigation of biotechnological potential of sponge-associated bacteria collected in Brazilian coast	Santos O.C.S., Soares A.R. et al.	2015
Discovery	Optimization of harvesting and extraction methods	Emerging biomedical applications of nano-chitins and nano-chitosans obtained via advanced eco-friendly technologies from marine resources	Muzzarelli R.A.A., El Mehtedi M. et al.	2014
Discovery	Optimization of harvesting and extraction methods	Extraction and applications of cyanotoxins and other cyanobacterial secondary metabolites	Haque F., Banayan S. et al.	2017
Discovery	Optimization of harvesting and extraction methods	Marine origin collagens and its potential applications	Silva T.H., Moreira-Silva J. et al.	2014
Discovery	Optimization of harvesting and extraction methods	Basic and recent advances in marine antihypertensive peptides: Production, structure-activity relationship and bioavailability	Abdelhedi O. & Nasri M.	2019
Discovery	Optimization of harvesting and extraction methods	MabCent: Arctic marine bioprospecting in Norway	Svenson J.	2013
Discovery	Optimization of harvesting and extraction methods	Antibacterial study of the seaweed Ulva fasciata	Archana G. & Sumathy V.J.H.	2019

Discovery	**Pharmacological analysis**	Role of marine macroalgae in plant protection & improvement for sustainable agriculture technology	Hamed S.M., El-Rhman A.A.A. et al.	2018
Discovery	Pharmacological analysis	Mechanisms for Pseudoalteromonas piscicidainduced killing of vibrios and other bacterial pathogens	Richards G.P., Watson M.A. et al.	2017
Discovery	Pharmacological analysis	Studies on biochemical and biological properties of turrids venom (Turricula javana and Lophiotoma indica)	Arumugam M., Giji S. et al.	2013
Discovery	Pharmacological analysis	Characterization and biotoxicity of Hypnea musciformis-synthesized silver nanoparticles as potential eco-friendly control tool against Aedes aegypti and Plutella xylostella	Roni M., Murugan K. et al.	2015
Discovery	Pharmacological analysis	Biotechnological Potential of Bacteria Isolated from the Sea Cucumber Holothuria leucospilota and Stichopus vastus from Lampung, Indonesia.	Wibowo J.Y., Kellermann M.Y. et al.	2019
Discovery	Pharmacological analysis	Thermophile-fermented compost extract as a possible feed additive to enhance fecundity in the laying hen and pig: Modulation of gut metabolism	Ito T., Miyamoto H. et al.	2016
Discovery	Pharmacological analysis	Identification and characterization of the antifungal substances of a novel streptomyces cavourensis NA4S	Pan H.Q., Yu S.Y. et al.	2015
Discovery	Pharmacological analysis	The antioxidant and anti-inflammatory effects of abalone intestine digest, Haliotis discus hannai in RAW 264.7 macrophages	Qian Z.J., Kim S.A. et al.	2012
Discovery	Pharmacological analysis	DNA damage protecting and free radical scavenging properties of mycosporine-2-glycine from the Dead Sea cyanobacterium in A375 human melanoma cell lines	Cheewinthamrongrod V., Kageyama H. et al.	2016
Discovery	Pharmacological analysis	Potential Anti-proliferative and Immunomodulatory Effects of Marine Microalgal Exopolysaccharide on Various Human Cancer Cells and Lymphocytes In Vitro	Park G.T., Go R.E. et al.	2017
Discovery	Pharmacological analysis	Hygrocin C from marine-derived Streptomyces sp. SCSGAA 0027 inhibits biofilm formation in Bacillus amyloliquefaciens SCSGAB0082 isolated from South China Sea gorgonian	Wang J., Nong X.H. et al.	2018
Discovery	Pharmacological analysis	Role of Myxicola infundibulum (Polychaeta, Annelida) mucus: From bacterial control to nutritional home site	Stabili L., Schirosi R. et al.	2014
Discovery	Pharmacological analysis	First insights into the biochemistry of Sabella spallanzanii (Annelida: Polychaeta) mucus: A potentially unexplored resource for applicative purposes	Stabili L., Schirosi R. et al.	2011
Discovery	Pharmacological analysis	Three-dimensional chitin-based scaffolds from Verongida sponges (Demospongiae: Porifera). Part II: Biomimetic potential and applications	Ehrlich H., Steck E. et al.	2010
Discovery	Pharmacological analysis	Antibacterial derivatives of marine algae: An overview of pharmacological mechanisms and applications	Shannon E. & Abu-Ghannam N.	2016
Discovery	Pharmacological analysis	The anti-rotaviral and anti-inflammatory effects of Hyrtios and Haliclona species	Koh S.I., Shin, H.S.et al.	2016
Discovery	Pharmacological analysis	Undaria pinnatifida fucoidan-rich extract recovers immunity of immunosuppressed mice	Lee H.H., Cho Y.J. et al.	2020
Discovery	Pharmacological analysis	A new abyssomicin polyketide with anti-influenza A virus activity from a marine-derived Verrucosispora sp. MS100137	Zhang J., Li B. et al.	2020
Discovery	Pharmacological analysis	Non-Cytotoxic Sulfated Heterorhamnan from Gayralia brasiliensis Green Seaweed Reduces Driver Features of Melanoma Metastatic Progression	Bellan D.L., Mazepa E. et al.	2020

Discovery	Pharmacological analysis	Anti-inflammatory effect of asterias amurensis fatty acids through NF-κB and MAPK pathways against LPS-stimulated RAW264.7 cells	Monmai C., Go S.H. et al.	2018
Discovery	Pharmacological analysis	Biological characterization of marine fish pathogen, Acinetobacter sp. strain An 2 producing antibacterial metabolites	Pandey A., Naik M.M. et al.	2011
Discovery	Pharmacological analysis	Isolation of Lactobacillus strains from shellfish for their potential use as probiotics	Kang C.H., Shyn Y.J. et al.	2016
Discovery	Pharmacological analysis	Algal fucoidan: Structural and size-dependent bioactivities and their perspectives	Morya V.K., Kim J. et al.	2012
Discovery	Pharmacological analysis	Nutrition, health, and disease: Role of selected marine and vegetal nutraceuticals	Corzo L., Fernandez-Novoa L. et al.	2020
Discovery	Pharmacological analysis	Anti-herpes simplex virus 1 and immunomodulatory activities of a poly-γ- glutamic acid from Bacillus horneckiae strain APA of shallow vent origin	Marino-Merlo F., Papaianni E. et al.	2017
Discovery	Pharmacological analysis	The cytotoxicity of dacarbazine potentiated by sea cucumber saponin in resistant B16F10 melanoma cells through apoptosis induction	Baharara J., Amini E. et al.	2016
Discovery	Pharmacological analysis	Evaluation of the anti-proliferative effects of Ophiocoma erinaceus methanol extract against human cervical cancer cells	Baharara J., Amini E. et al.	2016
Discovery	Pharmacological analysis	Thermophile-fermented compost as a possible scavenging feed additive toprevent peroxidation	Miyamoto H., Shimada E. et al.	2013
Discovery	Pharmacological analysis	Evaluation of wound healing and anti-inflammatory activity of a marine yellow pigmented bacterium, Micrococcus sp.	Srilekha V., Krishna G. et al.	2018
Discovery	Pharmacological analysis	Dictyopteris undulata extract induces apoptosis in human colon cancer cells	Kim A.D., Kang K.A. et al.	2014
Discovery	Pharmacological analysis	The p53 modulated cytotoxicity of Ophiocoma scolopendrina polysaccharide against resistance ovarian cancer cells	Amini E., Baharara J. et al.	2019
Discovery	Pharmacological analysis	Antiviral lead compounds from marine sponges	Sagar S., Kaur M. et al.	2010
Discovery	Pharmacological analysis	Cyanobacteria as nanogold factories: Chemical and anti-myocardial infarction properties of gold nanoparticles synthesized by lyngbya majuscula	Bakir E.M., Younis N.S. et al.	2018
Discovery	Pharmacological analysis	Ecklonia cava extract containing dieckol suppresses RANKL-Induced osteoclastogenesis via MAP Kinase/NF-κB pathway inhibition and heme oxygenase-1 induction	Kim S., Kang S.S. et al.	2019
Discovery	Pharmacological analysis	Evaluation of flavonoids from Zostera asiatica as antioxidants and nitric oxide inhibitors	Kim H., Jeong H. et al.	2016
Discovery	Pharmacological analysis	Antimicrobial compounds with therapeutic potential from Cerithidea cingulata against human and fish pathogens	Ashok Kumar P.	2011
Discovery	Pharmacological analysis	Effects of sulfated fucan, ascophyllan, from the brown Alga Ascophyllum nodosum on various cell lines: A comparative study on ascophyllan and fucoidan	Jiang Z., Okimura T. et al.	2010
Discovery	Pharmacological analysis	Biological activities of marine-derived oligosaccharides	Oda T.	2015
Discovery	Pharmacological analysis	Effect of phlorotannin-rich extracts of Ascophyllum nodosum and Himanthalia elongata (Phaeophyceae) on cellular oxidative markers in human HepG2 cells	Queguineur B., Goya L. et al.	2013
Discovery	Pharmacological analysis	K092A and K092B, two peptides isolated from the dogfish (Scyliorhinus canicula L.), with potential antineoplastic activity against human prostate and breast cancer cells	Bosseboeuf A., Baron A. et al.	2019

Discovery	Pharmacological analysis	Ascidians: An emerging marine model for drug discovery and screening	Dumollard R., Gazo I. et al.	2017
Discovery	Pharmacological analysis	Preparation and effects on neuronal nutrition of plasmenylethonoamine and plasmanylcholine from the mussel Mytilus edulis	Ding Y., Wang R. et al.	2020
Discovery	Pharmacological analysis	Sea cucumber Holothuria forskali, a new resource for aquaculture? Reproductive biology and nutraceutical approach	Santos R., Dias S. et al.	2016
Discovery	Pharmacological analysis	Marine actinobacterial metabolites and their pharmaceutical potential	Manivasagn P., Venkatesan J. et al.	2015
Discovery	Pharmacological analysis	Marine-derived penicillium species as producers of cytotoxic metabolites	Liu S., Su M. et al.	2017
Discovery	Pharmacological analysis	Structure, antiproliferative and cancer preventive properties of sulfated α-D-fucan from the marine bacterium Vadicella arenosi	Kokoulin M.S., Filshtein, A.P. et al.	2019
Discovery	Pharmacological analysis	Nutraceuticals from marine derived krill oil with immense heath potentials	Patel S.	2014
Discovery	Pharmacological analysis	Bioactive potential of actinobacteria isolated from the gut of marine fishes	Vignesh A., Ayswarya S. et al.	2019
Discovery	Pharmacological analysis	Nocardiopsis species: A potential source of bioactive compounds	Bennur T., Ravi Kumar A. et al.	2016
Discovery	Pharmacological analysis	Alginate-derived oligosaccharide inhibits neuroinflammation and promotes microglial phagocytosis of β-amyloid	Zhou R., Shi X.X. et al.	2015
Discovery	Pharmacological analysis	Perspectives on the use of marine and freshwater hydrobiont oils for development of drug delivery systems	Averina E.S. & Kutyrev I.A.	2011
Discovery	Pharmacological analysis	Amphidinol 22, a New Cytotoxic and Antifungal Amphidinol from the Dinoflagellate Amphidinium carterae	Martinez K.A., Lauritano C. et al.	2019
Discovery	Pharmacological analysis	Blue-print autophagy: Potential for cancer treatment	Ruocco N., Costantini S. et al.	2016
Discovery	Pharmacological analysis	Lysophosphatidylcholines and chlorophyll-derived molecules from the diatom cylindrotheca closterium with anti-inflammatory activity	Lauritano C., Helland K. et al.	2020
Discovery	Pharmacological analysis	Antagonistic effect of bacteria associated with ascidians from Thoothukudi coast	Mary M.R.P., Sugumar G. et al.	2016
Discovery	Pharmacological analysis	Vibrio anguillarum is genetically and phenotypically unaffected by long-term continuous exposure to the antibacterial compound tropodithietic acid	Rasmussen B.B., Grotkjær T. et al.	2016
Discovery	Pharmacological analysis	Identification and characterization of an antifungal protein, AFAFPR9, produced by marine-derived Aspergillus fumigatus R9	Rao Q., Guo W. et al.	2015
Discovery	Pharmacological analysis	The effects of supplemented diets with a phytopharmaceutical preparation from herbal and macroalgal origin on disease resistance in rainbow trout against Piscirickettsia salmonis	Hernandez A.J., Romero A. et al.	2016
Discovery	Pharmacological analysis	Marine Microalgae: Promising source for new bioactive compounds	De Vera C.R., Crespin C.D. et al.	2018
Discovery	Pharmacological analysis	Antibacterial, antifungal and antiprotozoal activities of fungal communities present in different substrates from Antarctica	Goncalves V.N., Carvalho C.R. et al.	2015
Discovery	Pharmacological analysis	From ecology to biotechnology, study of the defense strategies of algae and halophytes (from trapani saltworks, NW sicily) with a focus on antioxidants and antimicrobial properties	Messina C.M., Renda G. et al.	2019
Discovery	Pharmacological analysis	In vitro evaluation of antibacterial activity of Asparagopsis taxiformis from the Straits of Messina against pathogens relevant in aquaculture	Genovese G., Faggio C. et al.	2012

Discovery	Pharmacological analysis	The anti-biofilm activity secreted by a marine Pseudoalteromonas strain	Klein G.L., Soum-Soutéra E. et al.	2011
Discovery	Pharmacological analysis	Unravelling the antioxidant potential and the phenolic composition of different anatomical organs of the marine halophyte Limonium algarvense	Rodrigues M.J., Soszynski A. et al.	2015
Discovery	Pharmacological analysis	Selective inhibition of cancer cells' proliferation by compounds included in extracts from Baltic Sea cyanobacteria	Felczykowska A., Pawlik A. et al.	2015
Discovery	Pharmacological analysis	Large-scale bioprospecting of cyanobacteria, micro- and macroalgae from the Aegean Sea	Montalvao S., Demirel Z. et al.	2016
Discovery	Pharmacological analysis	Microbial Proteins as Novel Industrial Biotechnology Hosts to Treat Epilepsy	Amtul Z. & Aziz A.A.	2017
Discovery	Pharmacological analysis	Astaxanthin: Sources, extraction, stability, biological activities and its commercial applications - A review	Ambati R.R., Moi P.S. et al.	2014
Discovery	Pharmacological analysis	The Key Role of Sulfation and Branching on Fucoidan Antitumor Activity	Oliveira C., Ferreira A.S. et al.	2017
Discovery	Pharmacological analysis	Antibacterial properties of a glycolipid-rich extract and active principle from Nunavik collections of the macroalgae Fucus evanescens C. Agardh (Fucaceae)	Amiguet V.T., Jewell L.E. et al.	2011
Discovery	Pharmacological analysis	Semi-industrial Scale Production of a New Yeast with Probiotic Traits, Cryptococcus sp. YMHS, Isolated from the Red Sea.	El-Baz A., El-Enshasy H.A. et al.	2018
Discovery	Pharmacological analysis	Conventional and unconventional antimicrobials from fish, marine invertebrates and micro-algae	Smith V.J., Desbois A.P. et al.	2010
Discovery	Pharmacological analysis	Antimicrobial secondary metabolites from marine fungi: A mini review	Bajpai V.K.	2016
Discovery	Pharmacological analysis	Isolation and Antibiotic Screening of Fungi from a Hydrothermal Vent Site and Characterization of Secondary Metabolites from a Penicillium Isolate	Pan C., Shi Y. et al.	2017
Discovery	**Recombinant DNA technologies**	Cloning, expression, and characterization of a cold-active and organic solvent-tolerant lipase from aeromicrobium sp. SCSIO 25071	Su H., Mai Z. et al.	2016
Discovery	Recombinant DNA technologies	Recombinant mussel protein Pvfp-5β: A potential tissue bioadhesive	Santonocito R., Venturella F. et al.	2019
Discovery	Recombinant DNA technologies	RLj-RGD3, a novel recombinant toxin protein from Lampetra japonica, protects against cerebral reperfusion injury following middle cerebral artery occlusion involving the integrin-PI3K/Akt pathway in rats	Lu Q., Wang J. et al.	2016
Discovery	Recombinant DNA technologies	Characterization of a novel cold-adapted phosphinothricin N-acetyltransferase from the marine bacterium Rhodococcus sp. strain YM12	Wu G., Yuan M. et al.	2014
Discovery	Recombinant DNA technologies	Phycoerythrin-specific bilin lyase-isomerase controls blue-green chromatic acclimation in marine Synechococcus	Shukla A., Biswas A. et al.	2012
Discovery	Recombinant DNA technologies	A newly identified glutaminase-free L-Asparaginase (L-ASPG86) from the marine bacterium mesoflavibacter zeaxanthinifaciens	Lee S.Y., Lee Y. et al.	2016
Discovery	Recombinant DNA technologies	Self-encapsulation and controlled release of recombinant proteins using novel silica-forming peptides as fusion linkers	Abdelhamid M.A.A., Yeo K.B. et al.	2019
Discovery	Recombinant DNA technologies	Cloning, expression, and characterization of a cold-adapted and surfactant-stable alginate lyase from marine bacterium agarivorans sp. L11	Li S., Yang X. et al.	2015
Discovery	Recombinant DNA technologies	The adhesive properties of coacervated recombinant hybrid mussel adhesive proteins	Lim S., Choi Y.S. et al.	2010

Discovery	Recombinant DNA technologies	21: Blue biotechnology: a vision for future marine biorefineries	Prabha S.P., Nagappan S. et al.	2020
Discovery	Recombinant DNA technologies	Gene identification and characterization of fucoidan deacetylase for potential application to fucoidan degradation and diversification	Nagao T., Kumabe A. et al.	2017
Discovery	Recombinant DNA technologies	Identification and characterization of the fucoidanase gene from Luteolibacter algae H18	Nagao T., Arai Y. et al.	2018
Discovery	Recombinant DNA technologies	Molecular Cloning, Characterization, and Expression Analysis of a Prolyl 4-Hydroxylase from the Marine Sponge Chondrosia reniformis	Pozzolini M., Scarfì S. et al.	2015
Discovery	Recombinant DNA technologies	RHA-P: Isolation, expression and characterization of a bacterial α-L-rhamnosidase from Novosphingobium sp. PP1Y	De Lise F., Mensitieri F. te al.	2016
Discovery	Recombinant DNA technologies	Advances in time course extracellular production of human pre-miR-29b from Rhodovulum sulfidophilum	Pereira P., Pedro A.Q. et al.	2016
Discovery	Recombinant DNA technologies	Heterologous expression of the diazaquinomycin biosynthetic gene cluster	Braesel J., Tran T.A. et al.	2019
Discovery	Recombinant DNA technologies	Aii20J, a wide-spectrum thermostable N-acylhomoserine lactonase from the marine bacterium Tenacibaculum sp. 20J, can quench AHL-mediated acid resistance in Escherichia coli	Mayer C., Romero M. et al.	2015
Discovery	Recombinant DNA technologies	Silica particles with a quercetin-R5 peptide conjugate are taken up into HT-29 cells and translocate into the nucleus.	Del Favero G., Bialas F. et al.	2019
Discovery	**Screening**	Bioactive potential of seagrass bacteria against human bacterial pathogens	Ravikumar S., Thajuddin N. et al.	2010
Discovery	Screening	High throughput screening and profiling of high-value carotenoids from a wide diversity of bacteria in surface seawater	Asker D.	2018
Discovery	Screening	Isolation and characterization of Indole Acetic Acid (IAA) produced by a Halo tolerant marine bacterium isolated from coastal sand dune plants	Jayaprakashvel M., Abishamala K. et al.	2014
Discovery	Screening	Biodegradation of shrimp biowaste by marine Exiguobacterium sp. CFR26M and concomitant production of extracellular protease and antioxidant materials: production and process optimization by response surface methodology.	Anil Kumar P.K. & Suresh P.V.	2014
Discovery	Screening	Anti-MRSA and anti-TB metabolites from marine-derived Verrucosispora sp. MS100047	Huang P., Xie F. et al.	2016
Discovery	Screening	Production and characterization of a group of bioemulsifiers from the marine Bacillus velezensis strain H3	Liu X., Ren B. et al.	2010
Discovery	Screening	Screening of anti-biofilm compounds from marine-derived fungi and the effects of secalonic acid D on Staphylococcus aureus biofilm	Wang J., Nong X.H. et al.	2017
Discovery	Screening	Thermostable Bacteriocin BL8 from Bacillus licheniformis isolated from marine sediment	Smitha S. & Bhat S.G.	2013
Discovery	Screening	Isolation of a novel alginate lyase-producing Bacillus litoralis strain and its potential to ferment Sargassum horneri for biofertilizer.	Wang M., Chen L. et al.	2016
Discovery	Screening	A rapid and efficient screening method for antibacterial compound-producing bacteria	Hettiarachchi S.A., Lee S.J. et al.	2017

Discovery	Screening	Lipopeptide biosurfactants from Paenibacillus polymyxa inhibit single and mixed species biofilms	Quinn G.A., Maloy A.P. et al.	2012
Discovery	Screening	Bioprospecting from marine coastal sediments of Colombian Caribbean: screening and study of antimicrobial activity	Quintero M., Velasquez A. et al.	2018
Discovery	Screening	Two marine natural products, penicillide and verrucarin J, are identified from a chemical genetic screen for neutral lipid accumulation effectors in Phaeodactylum tricornutum	Yu M., Chen X. et al.	2020
Discovery	Screening	Isolation and initial characterization of a novel type of Baeyer-Villiger monooxygenase activity from a marine microorganism	Willetts A., Joint I. et al.	2012
Discovery	Screening	Characterization of an antimicrobial and antioxidant compound from a marine bacterium Gsa10 associated with the sponge Halichondria Glabrata	Phadale R. & Kumar M.S.	2018
Discovery	Screening	PTP1B inhibitory secondary metabolites from marine-derived fungal strains penicillium spp. And Eurotium sp.	Sohn J.H., Lee Y.R. et al.	2013
Discovery	Screening	Characterization and Biotechnological Potential Analysis of a New Exopolysaccharide from the Arctic Marine Bacterium Polaribacter sp. SM1127	Sun M.L., Zhao F. et al.	2015
Discovery	Screening	Bioactivity screening of microalgae for antioxidant, anti-inflammatory, anticancer, anti-diabetes, and antibacterial activities	Lauritano C., Handersen J.H. et al.	2016
Discovery	Screening	In vitro cholesterol-lowering properties of Lactobacillus plantarum AN6 isolated from aji-narezushi	Kuda T., Yazaki T. et al.	2013
Discovery	Screening	Evaluation of the biotechnological potential of bacterioplankton from Niterói coast, RJ	Jayme M.M.A., Castro R.O. et al.	2017
Discovery	Screening	Selection and evaluation of Malaysian Bacillus spp. strains as potential probiotics in cultured tiger grouper Epinephelus fuscoguttatus)	Yasin I.S.M., Razak N.F. et al.	2016
Discovery	Screening	In vitro antibacterial activity of sponge-associated fungi against bacterial aquaculture pathogens	Altunok M., Ozkaya F.C. et al.	2015
Discovery	Screening	Gram-Positive Marine Bacteria as a Potential Resource for the Discovery of Quorum Sensing Inhibitors	Teasdale M.E., Donovan K.A. et al.	2011
Discovery	Screening	Screening of marine Streptomyces spp. for potential use as probiotics in aquaculture	Das S., Ward L.R. et al.	2010
Discovery	Screening	Rapid screening of bioactive compounds from natural products by integrating 5-channel parallel chromatography coupled with on-line mass spectrometry and microplate based assays	Zhang Y., Xiao S. et al.	2013
Discovery	**Systematic literature review**	Some enzymes in marine environment: Prospective applications found in patent literature	Trincone A.	2012
Discovery	Systematic literature review	Global unbalance in seaweed production, research effort and biotechnology markets	Mazarrasa I., Olsen Y.S. et al.	2014
Discovery	Systematic literature review	A Bibliometric Analysis of Microalgae Research in the World, Europe, and the European Atlantic Area.	Rumin J., Nicolau E. et al.	2020
Discovery	Systematic literature review	A bibliometric-based analysis of the high-value application of Chlorella	Cheng Z., Kong W. et al.	2020
Discovery	Systematic literature review	Current knowledge on biotechnological interesting seaweeds from the Magellan Region, Chile	Mansilla A., Ávila M. et al.	2012

Discovery	Systematic literature review	Cnidarians as a source of new marine bioactive compounds - An overview of the last decade and future steps for bioprospecting	Rocha J., Peixe L. et al.	2011
Discovery	Systematic literature review	Carotenoids from Haloarchaea and Their Potential in Biotechnology.	Rodrigo-Banos M., Garbayo I. et al.	2015
**Productivity**	**Biocatalysis and biosynthetis**	Recent Progress in Chitosanase Production of Monomer-Free Chitooligosaccharides: Bioprocess Strategies and Future Applications	Sinha S., Chand S. et al.	2016
Productivity	Biocatalysis and biosynthetis	Thermophile-fermented compost as a fish feed additive modulates lipid peroxidation and free amino acid contents in the muscle of the carp, Cyprinus carpio	Tanaka R., Miyamoto H. et al.	2016
Productivity	Biochemical and molecular characterization	Biosynthetic origin of the carbon skeleton of a novel anti-tumor compound, haloroquinone, from a marine-derived fungus, Halorosellinia sp.	Niu C., Cai M. et al.	2012
Productivity	Biochemical and molecular characterization	Time-temperature-resolved functional and structural changes of phycocyanin extracted from Arthrospira platensis/Spirulina	Boker L., Hostettler T. et al.	2020
Productivity	Biochemical and molecular characterization	Sterilization of exopolysaccharides produced by deep-sea bacteria: impact on their stability and degradation.	Rederstoff E., Fatimi A. et al.	2011
Productivity	Biochemical and molecular characterization	Filamentous fungi are large-scale producers of pigments and colorants for the food industry	Dufossé L., Fouillaud M. et al.	2014
Productivity	Biochemical and molecular characterization	Lipid synthesized by micro-algae grown in laboratory- and industrial-scale bioreactors	Makri A., Bellou S. et al.	2011
Productivity	**Bioinformatics**	Modelling of multi-nutrient interactions in growth of the dinoflagellate microalga Protoceratium reticulatum using artificial neural networks	Lopez-Rosales L., Gallardo-Rodríguez J.J. et al.	2013
Productivity	**Chemical synthesis**	Marine derived polysaccharides as drug delivery systems	Awad G.A.S., Mortada N.D. et al.	2010
Productivity	Chemical synthesis	Antifreeze peptides and glycopeptides, and their derivatives: Potential uses in biotechnology	Bang J.K., Lee J. H. et al.	2013
Productivity	Chemical synthesis	Catechol-functionalized hydrogels: biomimetic design, adhesion mechanism, and biomedical applications.	Zhang W., Wang R. et al.	2020
Productivity	Chemical synthesis	Porous hydrogels from shark skin collagen crosslinked under dense carbon dioxide atmosphere	Fernandes-Silva S., Moreira-Silva J. et al.	2013
Productivity	**Omic technologies**	Reconstruction and analysis of the genome-scale metabolic model of schizochytrium limacinum SR21 for docosahexaenoic acid production.	Ye C., Qiao W. et al.	2015
Productivity	Omic technologies	Reconstruction and analysis of a genome-scale metabolic model of Nannochloropsis gaditana	Shah A.R., Ahmad A. et al.	2017
Productivity	Omic technologies	Comparative transcriptome analysis reveals that lactose acts as an inducer and provides proper carbon sources for enhancing exopolysaccharide yield in the deep-sea bacterium Zunongwangia profunda SM-A87	Qin Q.L., Li Y. et al.	2015
Productivity	Omic technologies	Fatty acid and metabolomic profiling approaches differentiate heterotrophic and mixotrophic culture conditions in a microalgal food supplement 'Euglena'.	Zeng M., Hao W. et al.	2016
Productivity	Omic technologies	Seafood traceability: Current needs, available tools, and biotechnological challenges for origin certification	Leal M.C., Pimentel T. et al.	2015
Productivity	**Optimization of culture conditions**	Pigment cell differentiation in sea urchin blastula-derived primary cell cultures.	Ageenko N.V., Kiselev K.V. et al.	2014

Productivity	Optimization of culture conditions	The effect of light, salinity, and nitrogen availability on lipid production by Nannochloropsis sp.	Pal D., Khozin-Goldberg I. et al.	2011
Productivity	Optimization of culture conditions	Optimization of polyhydroxybutyrate production by marine Bacillus megaterium MSBN04 under solid state culture	Sathiyanarayanan G., Kiran G.S. et al.	2013
Productivity	Optimization of culture conditions	Salicylic acid and its derivatives elicit the production of diterpenes and sterols in corals and their algal symbionts: a metabolomics approach to elicitor SAR	Farag M.A., Maamoun A.A. et al.	2018
Productivity	Optimization of culture conditions	Phycocyanin and phycoerythrin: Strategies to improve production yield and chemical stability	Hsieh-Lo M., Castillo G. et al.	2019
Productivity	Optimization of culture conditions	Nutrients from anaerobic digestion effluents for cultivation of the microalga Nannochloropsis sp. — Impact on growth, biochemical composition and the potential for cost and environmental impact savings	Mayers J.J., Ekman Nilsson A. et al.	2017
Productivity	Optimization of culture conditions	The preparation and characterization of a novel sphingan WL from marine Sphingomonas sp. WG	Li H., Jiao X. et al.	2016
Productivity	Optimization of culture conditions	Enhancement of astaxanthin production using Haematococcus pluvialis with novel LED wavelength shift strategy	Xi T., Kim D.G. et al.	2016
Productivity	Optimization of culture conditions	Mixed fermentation of Spirulina platensis with Lactobacillus plantarum and Bacillus subtilis by random-centroid optimization.	Bao J., Zhang X. et al.	2018
Productivity	Optimization of culture conditions	Fed-Batch Strategies for Production of PHA Using a Native Isolate of Halomonas venusta KT832796 Strain.	Stanley A., Kumar H.N.P. et al.	2018
Productivity	Optimization of culture conditions	Integrated process of two stage cultivation of Nannochloropsis sp. for nutraceutically valuable eicosapentaenoic acid along with biodiesel.	Mitra M., Patidar S.K. et al.	2015
Productivity	Optimization of culture conditions	Bioprocess development for the production of sonorensin by Bacillus sonorensis MT93 and its application as a food preservative	Chopra L., Singh G. et al.	2015
Productivity	Optimization of culture conditions	Saccharina japonica, a potential feedstock for pigment production using submerged fermentation	General T., Prasad B. et al.	2014
Productivity	Optimization of culture conditions	Ammonium acetate supplement strategy for enhancement of chaetominine production in liquid culture of marine-derived aspergillus fumigatus CY018	Liu C., Wei X. et al.	2019
Productivity	Optimization of culture conditions	Improvement of Curvulamine Production by Precursors Co-addition Strategy in Liquid Culture of Marine-Derived Fungus Curvularia sp. IFB-Z10.	Wei X.C, Liu C.Q. et al.	2020
Productivity	Optimization of culture conditions	Optimization of nutrients for dinactin production by a marine Streptomyces sp. from the high latitude Arctic	Zhou J., He H. et al.	2015
Productivity	Optimization of culture conditions	pH-Dependent accumulation of anticancer compound on mycelia in fermentation of marine fungus	Zhou W., Cai M. et al.	2014
Productivity	Optimization of culture conditions	Aquaculture of marine non-food organisms: what, why and how?	Leal M.C., Rocha R.J.M. et al.	2018
Productivity	Optimization of culture conditions	Biomass, lipid productivities and fatty acids composition of marine Nannochloropsis gaditana cultured in desalination concentrate.	Matos A.P., Feller R. et al.	2015
Productivity	Optimization of culture conditions	Fed-batch strategy for enhancing cell growth and C-phycocyanin production of Arthrospira (Spirulina) platensis under phototrophic cultivation	Xie Y., Jin Y. et al.	2015

Productivity	Optimization of culture conditions	Bioreactor technology in marine microbiology: From design to future application	Zhang Y., Arends J.B.A. et al.	2011
Productivity	Optimization of culture conditions	Statistical optimization of fermentation conditions and comparison of their influences on production of cellulases by a psychrophilic marine bacterium, psychrobacter aquimaris LBH-10 using orthogonal array method	Kim H.Y., Lee Y.J. et al.	2011
Productivity	Optimization of culture conditions	Optimization of culture conditions for penicilazaphilone C production by a marine-derived fungus Penicillium sclerotiorum M-22	Zhao H.G., Wang M. et al.	2018
Productivity	Optimization of culture conditions	Benefits of using algae as natural sources of functional ingredients.	Ibanez E. & Cifuentes A.	2013
Productivity	Optimization of culture conditions	Marine diatom Thalassiosira weissflogii based biorefinery for co-production of eicosapentaenoic acid and fucoxanthin	Marella T.K. & Tiwari A.	2020
Productivity	Optimization of culture conditions	Use of wavelength-selective optical light filters for enhanced microalgal growth in different algal cultivation systems.	Michael C., Del Ninno M. et al.	2015
Productivity	Optimization of culture conditions	A novel conico-cylindrical flask aids easy identification of critical process parameters for cultivation of marine bacteria	Mitra S., Sarkar S. et al.	2011
Productivity	Optimization of culture conditions	Biomass, Lipid and Fatty Acid Production in Large-Scale Cultures of the Marine Macroalga Derbesia tenuissima (Chlorophyta)	Magnusson M., Mata L. et al.	2014
Productivity	Optimization of culture conditions	A strategy for the highly efficient production of docosahexaenoic acid by Aurantiochytrium limacinum SR21 using glucose and glycerol as the mixed carbon sources	Li J., Liu R. et al.	2015
Productivity	Optimization of culture conditions	Production of fucoxanthin, chrysolaminarin, and eicosapentaenoic acid by Odontella aurita under different nitrogen supply regimes	Xia S., Gao B. et al.	2018
Productivity	Optimization of culture conditions	Production, characterization, and antioxidant activity of fucoxanthin from the marine diatom Odontella aurita.	Xia S., Wang K. et al.	2013
Productivity	Optimization of culture conditions	Biotechnological and pharmacological applications of biotoxins and other bioactive molecules from dinoflagellates	Assunao J., Catarina Guedes A. et al.	2017
Productivity	Optimization of culture conditions	Combined effects of nitrogen concentration and seasonal changes on the production of lipids in Nannochloropsis oculata.	Olofsson M., Lamela T. et al.	2014
Productivity	Optimization of culture conditions	On harnessing industrial waste groundnut oil cake as a low cost substrate for L-asparaginase production by marine-derived fungus aspergillus Niger AKV-MKBU	Vala A.K. & Dave B.P.	2019
Productivity	Optimization of culture conditions	Plastids of marine phytoplankton produce bioactive pigments and lipids	Heydarizadeh P., Poireir I. et al.	2013
Productivity	Optimization of culture conditions	Effects of Alginate Oligosaccharides on the Growth of Marine Microalgae	Ueno M. & Oda T.	2015
Productivity	Optimization of culture conditions	Enhancing lutein productivity of an indigenous microalga Scenedesmus obliquus FSP-3 using light-related strategies.	Ho S.H., Chan M.C. et al.	2014
Productivity	Optimization of culture conditions	Successes and pitfalls of the aquaculture of the sponge Mycale hentscheli	Page M.J., Handley S.J. et al.	2011
Productivity	Optimization of culture conditions	Enhanced production of Ca^{2+}-polymalate (PMA) with high molecular mass by Aureobasidium pullulans var. pullulans MCW.	Wang Y.K., Chi Z. et al.	2015
Productivity	Optimization of culture conditions	Enhancement of cell biomass and cell activity of astaxanthin-rich Haematococcus pluvialis	Sun H., Kong Q. et al.	2015

Productivity	Optimization of culture conditions	A Hetero-Photoautotrophic Two-Stage Cultivation Process for Production of Fucoxanthin by the Marine Diatom Nitzschia laevis.	Lu X., Sun H. et al.	2018
Productivity	Optimization of culture conditions	Lipid Production from Nannochloropsis.	Ma X.N., Chen T.P. et al.	2016
Productivity	Optimization of culture conditions	Enhancement of biomass, lipids, and polyunsaturated fatty acid (PUFA) production in Nannochloropsis oceanica with a combination of single wavelength light emitting diodes (LEDs) and low temperature in a three-phase culture system.	Sirisuk P., Sunwoo Y. et al.	2018
Productivity	Optimization of culture conditions	Biotechnological Production of Docosahexaenoic Acid Using Aurantiochytrium limacinum: Carbon Sources Comparison And Growth Characterization.	Abad S. & Turon X.	2015
Productivity	Optimization of culture conditions	Bacillamide C production by the optimized cultivation of the Bacillus atrophaeus strain C89 associated with the South China Sea sponge Dysidea avara	Jin L., Ma W. et al.	2011
Productivity	Optimization of culture conditions	Modification of artificial sea water for the mass production of (+)-terrein by Aspergillus terreus strain PF26 derived from marine sponge Phakellia fusca	Yin Y., Ding Y. et al.	2015
Productivity	Optimization of culture conditions	Optimization of fermentation conditions to enhance cytotoxic metabolites production by Bacillus velezensis strain RP137 from the persian gulf	Pournejati R., Karbalaei-Heidari H.R. et al.	2020
Productivity	Optimization of culture conditions	Algae for the production of SCP	Ghasemi Y., Rasoul-Amini S. et al.	2011
Productivity	Optimization of culture conditions	Optimization for enhanced production of antibacterial metabolites by marine actinomycetes Kocuria sp. Strain rsk4	Kumar R.R. & Jadeja V.J.	2017
Productivity	Optimization of culture conditions	Effect of light/dark cycles and pH on biomass of cyanobacteria (Anabaena ambigua) grown in an external-loop airlift photobioreactor	Venkataramanareddy M.B., Lakshmana Rao S.S. et al.	2016
Productivity	Optimization of culture conditions	Food Modulation Controls Astaxanthin Accumulation in Eggs of the Sea Urchin Arbacia lixula.	Galasso C., Orefice I. et al.	2018
Productivity	Optimization of culture conditions	Cultivation of the benthic microalga Prorocentrum lima for the production of diarrhetic shellfish poisoning toxins in a vertical flat photobioreactor.	Wang S., Chen J. et al.	2015
Productivity	Optimization of culture conditions	Microalgal biofactories: a promising approach towards sustainable omega-3 fatty acid production	Adarme-Vega T.C., Lim D.K.Y. et al.	2012
Productivity	Optimization of culture conditions	Mechanisms of fatty acid synthesis in marine fungus-like protists.	Xie Y. & Wang G.	2015
Productivity	Optimization of culture conditions	Modelling an Artificial Microalgae-Cyanobacteria Ecosystem	Caia M., Bernard O. et al.	2018
Productivity	Optimization of culture conditions	Macrocystis pyrifera source of nutrients for the production of carotenoids by a marine yeast Rhodotorula mucilaginosa	Leyton A., Flores L. et al.	2019
Productivity	Optimization of culture conditions	Production of eicosapentaenoic acid by Nannochloropsis oculata: Effects of carbon dioxide and glycerol.	Shene C., Chisti Y. et al.	2016
Productivity	Optimization of culture conditions	Influence of environmental variables in the efficiency of phage therapy in aquaculture	Silva Y.J., Costa L. et al.	2014
Productivity	Optimization of culture conditions	Bioreactors for microalgae: A review of designs, features and applications	Guedas A. & Malcata F.	2011
Productivity	Optimization of culture conditions	Effects of light and nitrogen availability on photosynthetic efficiency and fatty acid content of three original benthic diatom strains	Cointet E., Wielgosz-Collin G. et al.	2019
Productivity	Optimization of culture conditions	Polyunsaturated fatty acids in marine bacteria and strategies to enhance their production	Moi I.M., Leow A.T.C. et al.	2018

Productivity	Optimization of culture conditions	Effect of light quality supplied by light emitting diodes (LEDs) on growth and biochemical profiles of Nannochloropsis oculata and Tetraselmis chuii	Schulze P.S.C., Pereira H.G.C. et al.	2016
Productivity	Optimization of culture conditions	Bioactives from microalgal dinoflagellates.	Gallardo-Rodriguez J., Sánchez-Mirón A. et al.	2012
Productivity	Optimization of culture conditions	Pilot-scale outdoor photobioreactor culture of the marine dinoflagellate Karlodinium veneficum: Production of a karlotoxins-rich extract	Lopez-Rosales L., Sánchez-Mirón A. et al.	2018
Productivity	Optimization of culture conditions	System and method for research-scale outdoor production of microalgae and cyanobacteria	Schoepp N.G., Stewart R.L. et al.	2014
Productivity	Optimization of culture conditions	Vital parameters for biomass, lipid, and carotenoid production of thraustochytrids	Sohedein M.N.A., Wan-Mohtar W.A.A.Q.I. et al.	2020
Productivity	Optimization of culture conditions	Improvement in extracellular protease production by the marine antarctic yeast Rhodotorula mucilaginosa L7	Chaud L.C.S., Lario L.D. et al.	2016
Productivity	Optimization of culture conditions	Nitrogen or phosphorus repletion strategies for enhancing lipid or carotenoid production from Tetraselmis marina	Dahmen-Ben Moussa I., Chtourou H. et al.	2017
Productivity	Optimization of culture conditions	Thraustochytrids Can Be Grown in Low-Salt Media Without Affecting PUFA Production	Shabala L., McMeekin T. et al.	2013
Productivity	Optimization of culture conditions	Study of macro algae for marine biotechnology material from large scale offshore cultivation from multiple mooring system of large aquaculture ocean floating structure	Sulaiman O.O., Kader A.S.A. et al.	2013
Productivity	Optimization of culture conditions	Optimization of culture conditions and squalene enrichment from heterotrophic marine microalga Schizochytrium mangrovei PQ6 for squalene production	Anh H.T.L., Ha N.C. et al.	2016
Productivity	Optimization of culture conditions	Production of Calcaride A by Calcarisporium sp. in Shaken Flasks and Stirred Bioreactors.	Tamminen A., Wang Y. et al.	2015
Productivity	Optimization of culture conditions	Phycobiliprotein: Potential microalgae derived pharmaceutical and biological reagent	Manirafasha E., Ndikubwimana T. et al.	2016
Productivity	Optimization of culture conditions	The innovation of technology for microalgae cultivation and its application in functional foods and the nutraceutical industry	Satoh A., Ishikura M. et al.	2010
Productivity	Optimization of culture conditions	Optimization of the culture condition for an antitumor bacterium Serratia proteamacula 657 and identification of the active compounds	Miao L., Wang X. et al.	2013
Productivity	Optimization of culture conditions	Optimization of culture media for large-scale lutein production by heterotrophic Chlorella vulgaris.	Jeon J.Y., Kwon J.S. et al.	2014
Productivity	Optimization of culture conditions	Enhancing production of a 24-membered ring macrolide compound by a marine bacterium using response surface methodology.	Chen H., Wu M. et al.	2013
Productivity	**Optimization of harvesting and extraction methods**	Ultrasonic-assisted production of antioxidative polysaccharides from Crassostrea hongkongensis.	Cai B., Pan J. et al.	2014
Productivity	Optimization of harvesting and extraction methods	Integration of protein extraction with a stream of byproducts from marine macroalgae: A model forms the basis for marine bioeconomy	Gajaria T.K., Suthar D. et al.	2017
Productivity	Optimization of harvesting and extraction methods	Comparison between gelatines extracted from mackerel and blue whiting bones after different pre-treatments.	Khiari Z., Rico D. et al.	2013

Productivity	Optimization of harvesting and extraction methods	Purification of the exopolysaccharide produced by Alteromonas infernus: identification of endotoxins and effective process to remove them	Du A.G.L., Zykwinska A. et al.	2017
Productivity	Optimization of harvesting and extraction methods	Efficient harvesting of marine Chlorella vulgaris microalgae utilizing cationic starch nanoparticles by response surface methodology	Bayat Tork M., Khalilzadeh R. et al.	2017
Productivity	Optimization of harvesting and extraction methods	The influence of processing on the DNA integrity in several raw materials of marine foods	Xiao Y., Xuejiao Z. et al.	2015
Productivity	Optimization of harvesting and extraction methods	Pressurized fluid extraction of polyunsaturated fatty acids from the microalga Nannochloropsis oculata	Pieber S., Schober S. et al.	2012
Productivity	Optimization of harvesting and extraction methods	Antimicrobial activity of extracts from macroalgae Ulva lactuca against clinically important Staphylococci is impacted by lunar phase of macroalgae harvest	Deveau A.M., Miller-Hope Z. et al.	2016
Productivity	Optimization of harvesting and extraction methods	Cationic polymers for successful flocculation of marine microalgae	't Lam G.P., Vermue M.H. et al.	2014
Productivity	**Pharmacological analysis**	Dietary administration of the extract of Rhodobacter sphaeroides WL-APD911 enhances the growth performance and innate immune responses of seawater red tilapia (Oreochromis mossambicus×Oreochromis niloticus)	Chiu K.H., Liu W.S. et al.	2014
Productivity	Pharmacological analysis	Nutritional Potential and Toxicological Evaluation of Tetraselmis sp. CTP4 Microalgal Biomass Produced in Industrial Photobioreactors.	Pereira H., Silva J. et al.	2019
Productivity	**Recombinant DNA technologies**	Nitroreductase-mediated Gonadal Dysgenesis for Infertility Control of Genetically Modified Zebrafish	Hu S.Y., Lin P.Y. et al.	2010
Productivity	Recombinant DNA technologies	Importance of polyunsaturated fatty acids from marine algae	Zarate R., Jaber-Vazdekis N. et al.	2016
Productivity	Recombinant DNA technologies	The biological role of pituitary adenylate cyclase-activating polypeptide (PACAP) in growth and feeding behavior in juvenile fish	Lugo J.M., Oliva A. et al.	2010
Productivity	Recombinant DNA technologies	Advances in genetic engineering of marine algae.	Qin S., Lin H. et al.	2012
Productivity	Recombinant DNA technologies	Molecular cloning and characterization of the myostatin gene in a cultivated variety of bay scallop, Argopecten irradians	Guo L., Li L. et al.	2012
Productivity	Recombinant DNA technologies	Cyanobacteria: Review of current potentials and applications	Zahra Z., Choo D.H. et al.	2020
Productivity	Recombinant DNA technologies	Towards the industrial production of omega-3 long chain polyunsaturated fatty acids from a genetically modified diatom phaeodactylum tricornutum	Hamilton M.L., Warwick J. et al.	2015
Productivity	Recombinant DNA technologies	Chapter 12: Marine biotechnology for food	Joseph I. & Augustine A.	2020
Productivity	Recombinant DNA technologies	Production and isolation of azaspiracid-1 and -2 from Azadinium spinosum culture in pilot scale photobioreactors.	Jauffrais T., Kilcoyne J. et al.	2012
Productivity	Recombinant DNA technologies	Marine biotechnology advances towards applications in new functional foods	Freitas A.C., Rodrigues D. et al.	2012
Productivity	Recombinant DNA technologies	Immobilized sialyltransferase fused to a fungal biotin-binding protein: Production, properties, and applications	Kajiwara H., Tsunashima M. et al.	2016
Productivity	Recombinant DNA technologies	Isolation of Novel Exo-type β-Agarase from Gilvimarinus chinensis and High-level Secretory Production in Corynebacterium glutamicum	Jeong Y.J., Choi J.W. et al.	2019
Productivity	Recombinant DNA technologies	Biotechnological production of astaxanthin with Phaffia rhodozyma/Xanthophyllomyces dendrorhous	Schmidt I., Schewe H. et al.	2011

Productivity	Recombinant DNA technologies	Molecular cloning, over-expression and enzymatic characterization of an endo-acting β-1,3-glucanase from marine bacterium Mesoflavibacter zeaxanthinifaciens S86 in Escherichia coli	Lee Y., Lee J.H. et al.	2014
Productivity	Recombinant DNA technologies	Molecular cloning, overexpression, and enzymatic characterization of glycosyl hydrolase family 16 β-agarase from marine bacterium saccharophagus sp. AG21 in escherichia coli	Lee Y., Oh C. et al.	2013
Productivity	Recombinant DNA technologies	Enhancing production of microalgal biopigments through metabolic and genetic engineering.	Saini D.K., Chakdar H. et al.	2020
Productivity	Recombinant DNA technologies	Cyanobacterial pigments: Perspectives and biotechnological approaches.	Saini D.K., Pabbi S. et al.	2018
Productivity	Recombinant DNA technologies	Deleting the first disulphide bond in an arenicin derivative enhances its expression in Pichia pastoris	Yang N., Wang X. et al.	2017
Productivity	Recombinant DNA technologies	Molecular cloning, characterization, and heterologous expression of a new κ-carrageenase gene from marine bacterium Zobellia sp. ZM-2	Liu Z., Li G. et al.	2013
Productivity	Recombinant DNA technologies	Enhanced β-galactosidase production from whey powder by a mutant of the psychrotolerant yeast Guehomyces pullulans 17-1 for hydrolysis of lactose.	Xu J.L., Zhao J. et al.	2012
Productivity	Recombinant DNA technologies	Metabolic engineering for the microbial production of marine bioactive compounds	Mao X., Liu Z. et al.	2017
Productivity	Recombinant DNA technologies	CRISPR–Cas9 System for Genome Engineering of Photosynthetic Microalgae	Patel V.K., Soni N. et al.	2019
Productivity	Recombinant DNA technologies	The challenge of ecophysiological biodiversity for biotechnological applications of marine microalgae.	Barra L., Chandrasekaran R. et al.	2014
Productivity	Recombinant DNA technologies	Approaches for delaying sexual maturation in salmon and their possible ecological and ethical implications	Iversen M., Myhr A.I. et al.	2016
Productivity	Recombinant DNA technologies	Hepatocyte Nuclear Factor 4α Transactivates the Mitochondrial Alanine Aminotransferase Gene in the Kidney of Sparus aurata	Saldago M.C., Meton I. et al.	2012
Productivity	Recombinant DNA technologies	Immobilized Growth of the Peridinin-Producing Marine Dinoflagellate Symbiodinium in a Simple Biofilm Photobioreactor	Benstein R.M., Cebi Z. et al.	2014
Productivity	Recombinant DNA technologies	The lipid metabolism in thraustochytrids	Morabito C., Bournaud C. et al.	2019
Productivity	Recombinant DNA technologies	Microalgae in biotechnological application: A commercial approach	Khatoon N. & Pal R.	2015
Productivity	Recombinant DNA technologies	Characterization and Application of Marine Microbial Omega-3 Polyunsaturated Fatty Acid Synthesis.	Allemann M.N. & Allen E.E.	2018
Productivity	Recombinant DNA technologies	Marine chitinolytic enzymes, a biotechnological treasure hidden in the ocean?	Beygmoradi A., Homaei A. et al.	2018
Productivity	Recombinant DNA technologies	Developing diatoms for value-added products: Challenges and opportunities	Fu W., Wichuk K. et al.	2015
Productivity	Recombinant DNA technologies	Production of mannosylglycerate in Saccharomyces cerevisiae by metabolic engineering and bioprocess optimization	Faria C., Borges N. et al.	2018
Productivity	Recombinant DNA technologies	LodB is required for the recombinant synthesis of the quinoprotein l-lysine-ε-oxidase from Marinomonas mediterranea	Chacon-Verdu M.D., Gomez D. et al.	2014
Productivity	Recombinant DNA technologies	Development of Synechocystis sp. PCC 6803 as a phototrophic cell factory.	Yu Y., You L. et al.	2013

Productivity	Recombinant DNA technologies	Combinatorial biosynthesis of Synechocystis PCC6803 phycocyanin holo-α-subunit (CpcA) in Escherichia coli and its activities	Yu P., Li P. et al.	2016
Productivity	**Screening**	Culturable Diversity and Lipid Production Profile of Labyrinthulomycete Protists Isolated from Coastal Mangrove Habitats of China.	Wang Q., Ye H. et al.	2019
**Sustainability**	**Biocatalysis and biosynthetis**	Biosynthesis of fluorescent gold nanoparticles using an edible freshwater red alga, Lemanea fluviatilis (L.) C.Ag. and antioxidant activity of biomatrix loaded nanoparticles.	Sharma B., Purkayastha D.D. et al.	2014
Sustainability	Biocatalysis and biosynthetis	Anti-neoplastic selenium nanoparticles from Idiomarina sp. PR58-8	Srivastava P. & Kowshik, M.	2016
Sustainability	Biocatalysis and biosynthetis	Bioconversion of Chitin to Bioactive Chitooligosaccharides: Amelioration and Coastal Pollution Reduction by Microbial Resources	Kumar M., Brar A. et al.	2018
Sustainability	Biocatalysis and biosynthetis	Enzymatic routes for the production of mono- and di-glucosylated derivatives of hydroxytyrosol	Trincone A., Pagnotta E. et al.	2012
Sustainability	Biocatalysis and biosynthetis	Chitooligomers preparation by chitosanase produced under solid state fermentation using shrimp by-products as substrate.	Nidheesh T., Pal G.K. et al.	2015
Sustainability	Biocatalysis and biosynthetis	Valorisation of smooth hound (Mustelus mustelus) waste biomass through recovery of functional, antioxidative and antihypertensive bioactive peptides	Sayari N., Sila A. et al.	2016
Sustainability	Biocatalysis and biosynthetis	Rationale behind the near-ideal catalysis of Candida antarctica lipase A (CAL-A) for highly concentrating ω-3 polyunsaturated fatty acids into monoacylglycerols.	He Y., Li J. et al.	2017
Sustainability	Biocatalysis and biosynthetis	Characterization of silver nanoparticles synthesized by using marine isolate Streptomyces albidoflavus	Prakasham R.S., Kumar B.S. et al.	2012
Sustainability	Biocatalysis and biosynthetis	Extracellular Synthesis and Characterization of Gold Nanoparticles Using Mycobacterium sp. BRS2A-AR2 Isolated from the Aerial Roots of the Ghanaian Mangrove Plant, Rhizophora racemosa	Camas M., Camas A.Z. et al.	2018
Sustainability	Biocatalysis and biosynthetis	Characterization, antimicrobial and antioxidant property of exopolysaccharide mediated silver nanoparticles synthesized by Streptomyces violaceus MM72	Sivasankar P., Seedevi P. et al.	2018
Sustainability	Biocatalysis and biosynthetis	Green synthesis of gold nanoparticles by the marine microalga Tetraselmis suecica.	Shakibaie M., Forootanfar H. et al.	2010
Sustainability	Biocatalysis and biosynthetis	Phyco-linked vs chemogenic magnetite nanoparticles: Route selectivity in nano-synthesis, antibacterial and acute zooplanktonic responses	Mashjoor S., Yousefzadi M. et al.	2019
Sustainability	**Biochemical and molecular characterization**	Seaweeds: A resource for marine bionanotechnology	Vijayan S.R., Santhiyagu P. et al.	2016
Sustainability	Biochemical and molecular characterization	Discolored Red Seaweed Pyropia yezoensis with Low Commercial Value Is a Novel Resource for Production of Agar Polysaccharides	Sasuga K., Yamanashi T. et al.	2018
Sustainability	Biochemical and molecular characterization	Pollutants from fish feeding recycled for microalgae production as sustainable, renewable and valuable products	Chan H.	2019
Sustainability	Biochemical and molecular characterization	Bryostatins: Biological context and biotechnological prospects	Trindade-Silva A.E., Lim-Fong G.E. et al.	2010
Sustainability	Biochemical and molecular characterization	Xylanases from marine microorganisms: A brief overview on scope, sources, features and potential applications	Qeshmi F.I., Homaei A. et al.	2020

Sustainability	**Bioinformatics**	Environmental evaluation of eicosapentaenoic acid production by Phaeodactylum tricornutum	Perez-Lopez P., González-García S. et al.	2014
Sustainability	**Chemical synthesis**	The first total synthesis of the cyclodepsipeptide pipecolidepsin A	Pelay-Gimeno M., García-Ramos Y. et al.	2013
Sustainability	Chemical synthesis	Zinc oxide nanorod clusters deposited seaweed cellulose sheet for antimicrobial activity	Bhutiya P.L., Mahajan M.S. et al.	2018
Sustainability	**Drug discovery**	Towards sustainable sources for omega-3 fatty acids production	Adarme-Vega T.C., Thomas-Hall S.R. et al.	2014
Sustainability	**Omic technologies**	Bioactive secondary metabolites from octocoral-Associated microbes—New chances for blue growth	Raimundo I., Silva S.G. et al.	2018
Sustainability	**Optimization of culture conditions**	Highlights of marine invertebrate-derived biosynthetic products: their biomedical potential and possible production by microbial associants.	Radjasa O.K., Vaske Y.M. et al.	2011
Sustainability	Optimization of culture conditions	Economic evaluation of the commercial production between Brazilian samphire and whiteleg shrimp in an aquaponics system	Castilho-Barros L., Almeida F.H. et al.	2018
Sustainability	Optimization of culture conditions	Primary cell culture from the nose of a marine organism, the banded houndshark, Triakis scyllium	Jung S.M., Kim D.S. et al.	2013
Sustainability	Optimization of culture conditions	Sustainable production of biologically active molecules of marine based origin	Murray P.M., Moane S. et al.	2013
Sustainability	Optimization of culture conditions	Energy Budget for the Cultured, Zooxanthellate Octocoral Sinularia flexibilis	Khalesi M.K., Beeftink H.H. et al.	2011
Sustainability	Optimization of culture conditions	Evaluation of anaerobic digestates from different feedstocks as growth media for Tetradesmus obliquus, Botryococcus braunii, Phaeodactylum tricornutum and Arthrospira maxima.	Massa M., Buono S. et al.	2017
Sustainability	Optimization of culture conditions	Coral aquaculture to support drug discovery	Leal M.C., Calado R. et al.	2013
Sustainability	Optimization of culture conditions	Exploring marine resources for bioactive compounds	Kiuru P., Valeria D'Auria M. et al.	2014
Sustainability	Optimization of culture conditions	Cultivation of Nannochloropsis for eicosapentaenoic acid production in wastewaters of pulp and paper industry.	Polishchuk A., Valev D. et al.	2015
Sustainability	Optimization of culture conditions	Cultivation of Sponges, Sponge Cells and Symbionts. Achievements and Future Prospects	Schippers K.J., Sipkema D. et al.	2012
Sustainability	**Optimization of harvesting and extraction methods**	Conventional and alternative technologies for the extraction of algal polysaccharides	Hernandez-Carmona G., Freile-Pelegrín Y. et al.	2013
Sustainability	Optimization of harvesting and extraction methods	Environmentally Friendly Valorization of Solieria filiformis (Gigartinales, Rhodophyta) from IMTA Using a Biorefinery Concept.	Penuela A., Robledo D. et al.	2018
Sustainability	Optimization of harvesting and extraction methods	Diatom milking: a review and new approaches.	Vinayak V., Manoylov K.M. et al.	2015
Sustainability	Optimization of harvesting and extraction methods	Simple Method for Preparation of Nanostructurally Organized Spines of Sand Dollar Scaphechinus mirabilis (Agassiz, 1863)	Ehrlich H., Elkin Y.N. et al.	2011
Sustainability	Optimization of harvesting and extraction methods	Turning waste into usable products: A case study of extracting Chitosan from Blue Crab	Webster C., Onokpise O. et al.	2014

Sustainability	Optimization of harvesting and extraction methods	Isolation of an unusual metabolite 2-allyloxyphenol from a marine actinobacterium, its biological activities and applications	Arumugam M., Mitra A. et al.	2010
Sustainability	Optimization of harvesting and extraction methods	Chondroitin sulfate, hyaluronic acid and chitin/chitosan production using marine waste sources: Characteristics, applications and eco-friendly processes: A review	Vazquez J.A., Rodríguez-Amado I. et al.	2013
Sustainability	Optimization of harvesting and extraction methods	Innovative approach to sustainable marine invertebrate chemistry and a scale-up technology for open marine ecosystems	Vlachou P., Le Goffe G. et al.	2018
Sustainability	Optimization of harvesting and extraction methods	Clean bio-technologies for obtaining new pharmaceutical formulations based on collagen gels and marine algae extracts for medical applications	Sirbu R., Zaharia T. et al.	2010
Sustainability	Optimization of harvesting and extraction methods	Isolation and characterization of collagen from marine fish (Thunnus obesus)	Jeong H.S., Venkatesan J. et al.	2013
Sustainability	Optimization of harvesting and extraction methods	Innovative alternative technologies to extract carotenoids from microalgae and seaweeds	Poojary M.M., Barba F.G. et al.	2016
Sustainability	Optimization of harvesting and extraction methods	Microalgae essential oils	Galarza J. & Aguaiza C.	2018
Sustainability	Optimization of harvesting and extraction methods	Fish processing waste: A promising source of type-I collagen	Silvipriya K.S., Krishna Kumar K. et al.	2016
Sustainability	Optimization of harvesting and extraction methods	Marine collagen from alternative and sustainable sources: Extraction, processing and applications	Coppola D., Oliviero M. et al.	2020
Sustainability	Optimization of harvesting and extraction methods	Biotechnological Applications for the Sustainable Use of Marine By-products: In Vitro Antioxidant and Pro-apoptotic Effects of Astaxanthin Extracted with Supercritical CO2 from Parapeneus longirostris	Messina C.M., Manuguerra S. et al.	2019
Sustainability	Optimization of harvesting and extraction methods	Chapter 7: Fish Discards as Source of Health-Promoting Biopeptides	Perez-Galvez R., Espejo-Carpio F.J. et al.	2018
Sustainability	**Pharmacological analysis**	Nutraceuticals and bioactive compounds from seafood processing waste	Menon V.V. & Lele S.S.	2015
Sustainability	Pharmacological analysis	Exploring the valuable carotenoids for the large-scale production by marine microorganisms	Torregrosa-Crespo J., Montero Z. et al.	2018
Sustainability	Pharmacological analysis	Angiotensin I converting enzyme inhibitory peptides from fish by-products	Perez Galvez R., Espejo-Carpio F.J. et al.	2013
Sustainability	**Recombinant DNA technologies**	Efficient production of succinic acid from Palmaria palmata hydrolysate by metabolically engineered Escherichia coli	Olajuyin A.M., Yang M. et al.	2016
Sustainability	Recombinant DNA technologies	Aquatic biosystems: Applications in aquacultural engineering as a sustainable technology	Soto-Zarazua G.M., García-Trejo J.F. et al.	2014
